# Supplementary material for: Protocol for CARES–HAPIN: an ambidirectional cohort study on exposure to environmental tobacco smoke and risk of early childhood caries
Source: BMJ Open. 2024 May 15;14(5):e083874. doi: 10.1136/bmjopen-2024-083874 (PMC11097839; doi:10.1136/bmjopen-2024-083874)
Supplement: Author's manuscript [file bmjopen-2024-083874.draft_revisions.pdf]

BMJ Open is committed to open peer review. As part of this commitment we make the peer review history of every article we publish publicly available.

When an article is published we post the peer reviewers' comments and the authors' responses online. We also post the versions of the paper that were used during peer review. These are the versions that the peer review comments apply to.

The versions of the paper that follow are the versions that were submitted during the peer review process. They are not the versions of record or the final published versions. They should not be cited or distributed as the published version of this manuscript.

BMJ Open is an open access journal and the full, final, typeset and author-corrected version of record of the manuscript is available on our site with no access controls, subscription charges or pay-per-view fees (http://bmjopen.bmj.com).

If you have any questions on BMJ Open's open peer review process please email info.bmjopen@bmj.com

BMJ Open

Exposure to Environmental Tobacco Smoke and Risk of Early Childhood Caries: Design and Rationale of the CARES - HAPIN Study

Journal:	*BMJ Open*
Manuscript ID	bmjopen-2024-083874
Article Type:	Protocol
Date Submitted by the Author:	02-Jan-2024
Complete List of Authors:	Patil, Sneha; Sri Ramachandra Institute of Higher Education and Research (Deemed to be University), Department of Environmental Health Engineering, Faculty of Public Health; Dr. D.Y. Patil Dental College and Hospital, Dr. D.Y. Patil Vidyapeeth, Department of Pediatric and Preventive Dentistry<br>Puttaswamy, Naveen; Sri Ramachandra Institute of Higher Education and Research (Deemed to be University), Department of Environmental Health Engineering, Faculty of Public Health<br>Cardenas, Andres; Stanford School of Medicine, Stanford University, Department of Epidemiology and Population Health<br>Barr, Dana; Rollins School of Public Health, Emory University, Gangarosa Department of Environmental Health<br>Ghosh, Santu; St John's Medical College, Department of Biostatistics<br>Balakrishnan, Kalpana; Sri Ramachandra Institute of Higher Education and Research (Deemed to be University), Department of Environmental Health Engineering, Faculty of Public Health
Keywords:	PUBLIC HEALTH, Community child health < PAEDIATRICS, EPIDEMIOLOGIC STUDIES

SCHOLARONE™
Manuscripts

Original Research

Exposure to Environmental Tobacco Smoke and Risk of Early Childhood Caries: Design and Rationale of the CARES - HAPIN Study

Sneha S. Patil,[1,2] Naveen Puttaswamy,[1*] Andres Cardenas,[3] Dana Boyd Barr,[4] Santu Ghosh,[5] Kalpana Balakrishnan[1]

[1]Department of Environmental Health Engineering, Faculty of Public Health, Sri Ramachandra Institute of Higher Education and Research, Chennai, Tamil Nadu, India

[2] Department of Pediatric and Preventive Dentistry, Dr. D.Y. Patil Dental College and Hospital, Dr. D.Y. Patil Vidyapeeth, Sant-Tukaram Nagar, Pimpri, Pune, India

[3] Department of Epidemiology and Population Health, Stanford School of Medicine, Stanford University, Stanford, CA, USA

[4] Gangarosa Department of Environmental Health, Rollins School of Public Health, Emory University, Atlanta, GA, USA

[5] Department of Biostatistics, St. John's Medical College, Bengaluru, India

*Corresponding author

For peer review only

ABSTRACT

Introduction: Prenatal and postnatal exposure to environmental tobacco smoke (ETS) has been linked with early childhood caries (ECC), but the specific molecular mechanisms and pathways remain largely unknown. The Caries Risk from exposure to Environmental tobacco Smoke (CARES) within the Household Air Pollution Intervention Network (HAPIN) study aims to establish the association between ETS and ECC by employing epidemiologic and novel biomarker-based approaches. Here we outline the overall design and rationale of the project.

Methods: We will leverage the infrastructure and data from the prospective birth cohort (HAPIN trial, India) to mount the CARES study. Children (n=735, aged: 3 to 5 years) will undergo ECC examination by a trained dentist using standard criteria and calibrated methods. Structured questionnaires will be used to gather information on sociodemographic variables, dietary habits, oral hygiene, and current exposure to ETS. We will collect non-invasive or minimally invasive biospecimens (i.e., saliva, buccal cells, dried blood spots, and urine) from a subset of HAPIN children (n=120) to assess a battery of biomarkers indicative of exposure to ETS, early biological effect and epigenetic modifications. Both self-reported and objective measures of ETS exposure collected longitudinally at pre- and post-natal periods will be accessed from the HAPIN data. We will apply current science data techniques to assess the association and interrelationships between ETS, ECC, and multiple biomarkers.

Discussion: While there is compelling evidence that prenatal and postnatal ETS exposure promotes development of ECC, we are still yet to resolve the precise impact of these environmental exposures on the trajectory of dental caries. The findings generated from this study can further our understanding of biological underpinnings of ECC through modifiable environmental risk factors at the chemical level and the paramountcy of exposure timing. This could potentially improve the prevention strategies at a population level.

Keywords

Environmental tobacco smoke, early childhood caries, birth cohort, biomarkers, DNA methylation

Key Findings

What is already known about this topic?

Fetal and early life exposures to environmental chemicals and other exogenous factors mediate childhood oral health outcomes. Numerous studies have investigated the potential association of environmental tobacco smoke (ETS) exposure and early childhood caries (ECC), yielding conflicting results. A meta-analysis of 21 studies demonstrated that children exposed to ETS conferred greater odds [prenatal - OR:1.57 (1.47–1.67); postnatal - OR:1.72 (1.45–2.05)] of developing dental caries.

What this study adds?

To our knowledge, this will be the first study to delve into the underlying biological pathways that link ETS to ECC by applying multivariate statistics for in-depth analysis of high dimensional data. This proposed research will evaluate whether there is a specific exposure window or trimester of ETS associated with an increased susceptibility to childhood caries. Furthermore, it seeks to unveil the profound impact of ETS exposure on epigenetic

For peer review only

programming with potential implications for development of dental caries far into adulthood. Overall, this work would lay a foundation for future studies to better understand the interaction between modifiable environmental risk factors and impacts on other oral diseases.

How might this study affect research, practice or policy?

This study will provide evidence to help disseminate public health messages and assist in the development of community awareness programs addressing the negative effects of ETS exposure on dental caries in childhood, particularly in developing nations.

For peer review only

BACKGROUND

The World Health Organization estimates that approximately one-quarter of human diseases globally are attributable to environmental causes that can be obviated.[1] The first 1000 days of life (conception to 2 years), is a discrete window as it marks the beginning of rapid growth. Any disturbances due to environmental insults during this stage may have enduring impacts on health.[2,3] Environmental factors, inclusive of chemical contaminants, diet, lifestyle choices, social capital, stress, climate, and biological response to exposures collectively make up the exposome, the environmental complement of the genome.[4,5] The genetic code is largely static and non-modifiable, while the epigenome is modifiable and has garnered attention as a target for preventive strategies. This has led to an appreciation of the need to capture data from multiple *omics* layers using a data-driven approach to assess how environmental exposures influence individuals' health. Emerging evidence indicates that exposure to environmental toxicants such as environmental tobacco smoke (ETS) and metal(loid)s can modify oral health.[6-10]

Oral diseases affect an estimated 3.5 billion people worldwide, with dental caries standing out as the most prevalent condition.[11] The stark inequalities, unparalleled burden, and shared risk factors with other non-communicable diseases, underline dental caries as a major public health problem.[11] Understanding the role of environmental pollutants is imperative for disease prevention as oral health is an integral aspect of general health.[11] This concern is particularly relevant for early childhood caries (ECC), as it is largely preventable and five times as common as asthma.[12,13] ECC is a dynamic, multifactorial disease, driven by biofilm and sugar, leading to phasic demineralization and remineralization of tooth structure.[14] In recent years, social determinants of health have gained prominence as crucial drivers of ECC.[15] Furthermore, it is closely linked to the 17 Sustainable Development Goals and strongly connected to six of the thirteen health targets in goal 3 - Good Health and Well-being.[16] Globally, more than 600 million children have untreated dental caries in primary teeth.[14] This not only causes pain and infection but the ramifications extend to negatively impact children's growth, neurodevelopment, school performance, and quality of life - all culminating in tremendous encumbrance on the family and society.[13,17] Notably, ECC is on the rise in racially and ethnically minoritized groups, as well as those living in low- and middle-income nations. Among Indian children, the prevalence of ECC varies between 42% to 63%.[18] This disparity arises from limited access to dental care services, financial barriers, scarcity of dental professionals, and lack of awareness about oral health.[13,17]

For decades, researchers have meticulously studied the etiology of ECC and developed strategies to combat it. Despite this, its profound burden persists unabated. It may be prudent to explore environmental risk factors beyond the traditional factors in our quest to unravel this complex condition.

Mounting evidence evinces fetal and early-life exposure to ETS, even at low levels, increases the susceptibility of dental caries in children and adult life.[6-9] A recent meta-analysis has demonstrated that children residing with a smoker had significantly higher odds [prenatal - OR:1.57 (1.47–1.67); postnatal - OR:1.72 (1.45–2.05)] of presenting with dental caries.[7] ETS, an ubiquitous pollutant, also known as 'passive smoke' or 'secondhand smoke' refers to the mixture of smoke emitted from a burning tobacco product and exhaled by an active smoker.[19] According to the 2014 US Surgeon General's report, there are numerous detrimental health outcomes related to ETS exposure

For peer review only - http://bmjopen.bmj.com/site/about/guidelines.xhtml

including sudden infant death syndrome, preterm birth, impaired fetal growth, neurocognitive problems, cardiovascular diseases, and respiratory illness, all of which can persist into adulthood.[20] Recent systematic reviews and reports suggest that there is 'no safe level 'of ETS exposure.[19-22] In India, about 23 to 39% of the population is exposed to ETS, disproportionately affecting children and adolescent non-smokers.[23] The underlying mechanism linking ETS exposure to ECC pathogenesis remains poorly characterized, though several pathways including impaired salivary gland function, low levels of serum vitamin C, and enhanced streptococcus mutans proliferation are hypothesized to be involved.[24-26] Currently, questionnaires are commonly used for assessing pre and postnatal ETS exposures; however, they are inadequate for measuring chemical exposures and suffer from misclassification, recall bias and reporting bias. Moreover, assessing exposure cross-sectionally at a given time point only provides a limited understanding of associations and fails to capture the complete range of lifelong exposures along with limited correlational interpretation. However, epigenetics, particularly DNA methylation (DNAm), offers a potential solution as it allows for the retention of environmental perturbations over time. For example, smoking-associated DNAm markers serve as biomarkers of smoke exposure, including the prenatal period.[27] Additionally, DNA adducts originating from tobacco specific nitrosamines (TSNAs) can also be used as markers of cumulative ETS exposure and early biological effects.

As yet, in low-resourced settings such as India where exposure magnitudes and ECC prevalence are among the highest, evidence for the role of ETS exposure on dental caries in childhood is lacking. Even less is known about the independent and combined impact of ETS exposure during fetal development and/or early childhood on ECC occurrence. In addition, there have been no published studies that have examined variation in DNAm associated with ETS exposure and ECC. To address this gap, we will adopt epidemiological and mechanistic approaches to test the association between pre- and postnatal ETS exposure and ECC in HAPIN children in India. This research aligns with the National Institute of Dental and Craniofacial Research and the Indian National Oral Health Program's mission to enhance oral health. Unpacking the complexity of biological pathways involved in environmental pollutants and ECC development and the challenges endured by children and their parents will add a major step towards personalized, precision environmental health and precision dental care.

Study Aims:

Using an integrated approach that includes ECC evaluation, ETS exposure measurements, and early biological markers of effect and susceptibility, we aim to: i) determine whether *in-utero*, infant, and current exposures to ETS (self-reported and measured) are associated with stages of ECC; ii) assess select biomarkers in saliva, buccal cells, dried blood spot (DBS) and urine indicative of exposure to ETS, oxidative stress, inflammation, and epigenetic modifications. A conceptual framework underlying this study is shown in fig.1

METHODS

Study Setting and Participants

For peer review only - http://bmjopen.bmj.com/site/about/guidelines.xhtml

Caries Risk from exposure to Environmental tobacco Smoke (CARES) is an ancillary study within the HAPIN cohort in India. The HAPIN trial (NCT02944682) was launched in 2017 across four diverse countries (India, Rwanda, Guatemala, and Peru) to assess the health benefits of clean cooking fuel intervention on birth outcomes, pneumonia, and child growth. A detailed description of the HAPIN population and protocol has been previously outlined.[28] In brief, between May 2018 and February 2020, 800 pregnant women were recruited from the Kallakurichi (KK) and Nagapattinam (NP) districts in Tamil Nadu, India. The study enrolled women between the ages of 18-35 years during antenatal visits at Primary Health Centres in the region, selecting those with a viable singleton pregnancy between 9 to < 20 weeks gestation, who were Tamil-speaking, and planning to deliver and receive prenatal care visits at local community clinics or hospitals. Assessments were conducted periodically during enrollment in early pregnancy, 24-28 weeks gestation, 32-36 weeks gestation, and infancy (3 months, 6 months, and 12 months). The study continues to follow up with mothers and their children, with data collected bi-annually. Of the initial 773 live births at the time of delivery, the HAPIN cohort is currently monitoring 735 children, aged between 3 to 5 years, who will be eligible for inclusion. Kalrayan Hills, KK district is home to 372 children who live at an altitude of 800 m above sea level, while the blocks of Keezhvelur, Kezhayur, Thalainayiru, and Vedaranyam in NP, a coastal district, have 363 children residing at elevations ranging from 10 to 50 m above sea level.

Ethical Aspects and Consent

CARES study protocol has been approved by the institutional review board of the participating institute. Agreements for sharing materials and data will be formulated based on the preferred standards of the institution and HAPIN guidelines. The conduct of this study will align with ethical principles outlined in the Belmont Report and follow Good Clinical Practice guidelines in line with the Indian Council of Medical Research.

Written informed consent will be obtained from the parent(s)/legal guardian(s) of all children. Consent protocol entails providing participants with comprehensive information regarding the aims, methods, potential risks, and benefits of participation. Parents will be informed that their child's participation is voluntary, and even if they choose not to, their child will still receive the necessary care in accordance with HAPIN guidelines. Moreover, parents will be reassured that appropriate measures are in place to safeguard their children's personal information. They will be made aware that in the event an abnormality is detected during the analysis of samples, reports will be communicated to them, and further testing and medical interventions be advised as necessary.

Data Collection

Clinical Dental Examination

Dental caries assessment will be undertaken by visual examination using plain mouth mirrors, WHO CPI probe, and cotton rolls to remove debris and moisture when required. The diagnosis for each tooth surface will be based on modified International Caries Detection and Assessment (ICDAS) criteria; code 0 - indicates no evidence of caries, codes 1–2 initial lesions, codes 3–4 moderate, and 5–6 extensive caries lesions.[29] If a surface has multiple

For peer review only - http://bmjopen.bmj.com/site/about/guidelines.xhtml

lesions, it will be coded according to the most severe presentation. To determine individual caries experience, the percentage of affected surfaces out of all examined surfaces will be calculated. The intra-examiner reproducibility will be evaluated through two dental examinations conducted in 30 children at a 15-day interval by a trained and calibrated pediatric dentist. Additional clinical domains will encompass the evaluation of skeletal and occlusal characteristics, dental trauma, and developmental defects of the enamel.

Anthropometric Measurements

Children's height and weight will be measured by the trained surveyors. The US Centers for Disease Control and Prevention growth reference data will be used to compute body mass index, Z-scores and assign corresponding BMI categories (such as underweight, normal weight, overweight, and obese).

Exposure to ETS Assessment

All pregnant women recruited in the HAPIN India site are non-smokers and 32% reported exposure to ETS in the baseline survey. Extant data on measures of cotinine (COT), 3-hydroxycotinine (3OH COT), and polycyclic aromatic hydrocarbons (PAH) during pregnancy and early childhood (up to 1 year of the child) will be accessed from the HAPIN database. At the time of the ECC assessment, we will capture the smoking status of family members by administering a short questionnaire to the parent(s)/legal guardian(s). Both self-reported and objective measures of ETS exposure captured during prenatal and early childhood will be used to assess the risk of caries development. Additionally, in a subset of the children, COT, 3OH-COT, and PAH metabolites will be measured in saliva and urine to assess current exposure to ETS (details provided in the biomonitoring section).

Questionnaire

Trained bilingual staff members will interview parents to collect information via detailed questionnaires on oral hygiene (initiation of brushing, frequency, parental supervision, use of fluoride toothpaste usage), diet (frequency of sweets consumption, eating before sleep), dental attendance (dental visit frequency and reasons), and receipt of medications. The questionnaire has been designed by the research team referencing the American Academy of Pediatric Dentistry guidelines and pre-tested for clarity. Baseline data encompassing socioeconomic status, demographic details, household composition, pregnancy-related particulars, dietary diversity, and household expenses will be obtained from the HAPIN research electronic data capture server. Information related to the child such as age, gender, type of delivery, only child, and feeding patterns will also be abstracted.

Biomonitoring

Biomarker analyses are central component of this study, drawing on existing biomarker research associated with ETS exposure and health. Biomarkers related to tobacco smoke exposure, inflammation, oxidative stress, and epigenetic changes have been chosen to inform mechanistic pathways to link ETS exposure and ECC occurrence;

For peer review only - http://bmjopen.bmj.com/site/about/guidelines.xhtml

and also predict future dental caries risk (fig. 2). Standard protocols established for resource-limited field settings will be followed to ensure sample integrity when collecting biospecimens from children.[30]

Biological Matrix and Biomarker Selection

We will collect non-invasive or minimally invasive biospecimens including saliva, buccal cells, DBS, and urine from a subset (n=120) of the HAPIN children.

Saliva

Saliva is increasingly used as an alternative matrix for biomonitoring, particularly in children. Its collection is non-invasive, economical, easily accessible, and does not necessitate medical personnel in field settings with limited resources. Saliva provides recent physiological information about an individual through organic and inorganic components from the blood. Studies have shown a high correlation (r=0.94 to 0.99) in COT levels between saliva and plasma in both smokers and non-smokers.[31,32] Further, saliva is the preferred choice for assessing cariogenic bacteria counts and antioxidant status related to ECC. Saliva will be collected by passive drool from children ensuring no food or drink has been consumed 1h into the visit. They will be requested to sit still and silent in a comfortable position for 2-3 minutes. Saliva obtained in the first ten seconds will be discarded while that gathered thereafter within five minutes (approximately 1 to 2mL) will be pooled in a single collection vial. We will also collect saliva samples from mothers for correlation with COT, 3OH COT levels, and bacterial count assessments.

Buccal cells

Oral cell DNA is readily obtainable and constitutes a physiological domain impacted by ETS. ORAcollect pediatrics (OC-175, DNA Genotek, ON, Canada) DNA collection kits will be used to obtain buccal cells. The sponge tip will be positioned comfortably in the mouth and rubbed along both lower gums in a back-and-forth motion. After collection, the sponge tip will be placed into its corresponding tube containing the Oragene solution and gently mixed. Genomic DNA will be extracted with the prepIT•L2P kits from DNA Genotek and sample aliquots will be stored at −80°C until analysis.

Urine

Urine is a matrix of choice for environmental chemical metabolites that inform about biotransformation and internal exposure. Convenience or spot urine samples will be collected in sterile cups following ECC assessment. Urine will be aliquoted into cryo-chill vials and stored in deep freezers at -80°C until further analysis. A small aliquot will be used to measure creatinine using a semi-automatic biochemistry analyzer (Chem 7, Erba Mannheim, London UK) through spectrophotometry. Further, specific gravity will be measured with a urine refractometer PAL-10S (Atago Co. Ltd., Tokyo, Japan).

Dried blood spots (DBS)

We will collect routine blood samples as DBS on a five-spot Guthrie filter paper card (Whatman #903). This approach addresses the challenges associated with collecting, transporting, and storing venipuncture samples.[33] Also, DBS blood collection in children is minimally invasive, cost-effective, and requires low blood volume. In brief, the field staff will collect a DBS sample from the children via finger prick after hand warming and arm

For peer review only - http://bmjopen.bmj.com/site/about/guidelines.xhtml

massage to stimulate sufficient capillary blood flow for collection. The puncture site will be disinfected, and a sterile lancet will be used to puncture the ring or middle finger of the child's non-dominant hand. The first blood drop will be wiped away and subsequent blood drops will be applied to filter paper. Following sample collection, a sterile gauze pad will be pressed against the site until the bleeding stops. Samples will be air-dried for 24-48 hours and then placed in a biospecimen bag with desiccant and a humidity indicator card.[33,34]

Biomarker Measurements

The choice of biomarker under consideration in each matrix, the analytic technique, and its significance to the objectives of the CARES study is summarized in Table 1. The analytic protocol of each biomarker is provided in the sections below.

Biomarkers in Saliva

To evaluate the saliva flow rate, the collected saliva will be quantified in milliliters per minute, and pH levels checked within 30 minutes of collection. Salivary counts of Mutans streptococci and Lactobacilli will be determined using TaqMan PCR assay.[35] Total antioxidant capacity and reduced glutathione levels will be measured using the Ferric Reducing Antioxidant Power (FRAP) assay and by immunoassay technique, respectively.

Cotinine and 3-hydroxycotinine: The half-life of COT (~16 h) is longer compared to nicotine (~ 2h) and is a predominant and stable metabolite of nicotine in body fluids. A simple, rapid, and sensitive method will be used to measure COT and 3OH-COT simultaneously by the liquid chromatography-electrospray ionization-tandem mass spectrometry (LC-ESI-MS/MS) technique. An aliquot of saliva will be centrifuged in a cooling micro-centrifuge (Eppendorf 5430R) at 5000 RPM for 10 min. An equal volume of acetone will be added to the supernatant, with the final volume adjusted to 1mL using 0.1% formic acid in methanol solution. The resulting mixture will be vortex mixed and passed through Millex-HV syringe filters (4mm, 0.45µm) prior to analysis by LC-ESI-MS/MS. The limit of detection (LOD) for this method is 0.5 ng/mL, with a recovery of 99.6% and relative standard deviation (RSD) ranging from 9.2 to 9.5% for salivary COT in smoker's and non-smoker's samples.[36]

Metal(loid)s analysis: We will analyze trace metals in saliva and investigate their correlation with salivary COT to confirm if exposure to ETS contributes to the presence of metals in children. A recent study with 6-7-year-old children from the Family Life Project revealed positive correlations between salivary COT and Pb, Zn, and Cu, providing evidence for ETS as a source of trace metals.[37] Briefly, 0:5 mL of saliva will be weighted and digested overnight in 2 mL of ultrapure concentrated HNO3 acid. Subsequently, samples will be centrifuged at 15,000 rpm for 5min at 4°C. The supernatant will be diluted at a 1:100 ratio with 1% nitric acid with indium as the internal standard and metal concentrations will then be measured on a single run of triple quadrupole inductively coupled plasma mass spectrometer (QQQ-ICP-MS, Agilent™-8900). Quality control (QC) measures will be employed to ensure accurate concentration estimates: procedural blanks, replicates, initial and continuous calibration

For peer review only - http://bmjopen.bmj.com/site/about/guidelines.xhtml

For peer review only

verification. To assess the validity and reliability of measuring metals in the saliva of children, we will conduct a pilot analysis of paired samples (n=10) of saliva, venous blood, and DBS collected from healthy adult volunteers.

Malondialdehyde (MDA) and uric acid (UA): Measurement of MDA and UA is essential for studying mitochondrial functions as these components play a key role in energy metabolism and the production of reactive oxygen species. Saliva samples will be treated with perchloric acid, followed by centrifugation to precipitate large proteins. The resulting supernatant will be neutralized with potassium carbonate and filtered through a 0.22 μm filter. A 20 μL of the filtrate will be loaded for chromatographic analysis in a HPLC system with ultraviolet detection.[38]

Biomarkers in Buccal Cells

DNA methylation (DNAm): Buccal cell DNA will be used to analyze DNAm with the Infinium Methylation EPIC v2.0 BeadChip which targets over 935,000 CpG sites. To ensure QC, replicates and randomization of samples across chips and plates will be employed. DNAm data will be processed using the minfi R package.[39] Samples not meeting certain criteria (e.g., duplicate samples, poor individual call rate <0.98, or genotyping/sex mismatch) will be excluded. Background correction, dye bias adjustment through the 'noob' method, and quantile normalization will be performed according to established procedures.[40]

DNA adducts: We will focus on quantifying DNA adducts formed by TSNAs and PAHs using the LC-ESI-MS method as described elsewhere.[41,42] TSNAs such as 4-(N-nitrosomethylamino)-1-(3-pyridyl)-1-butanone (NNK) and N′-nitrosonornicotine (NNN) are found together in environments contaminated by tobacco smoke. The metabolic process involving α-hydroxylation at the 2'-position of NNN or the methyl group of NNK leads to the formation of a reactive intermediate (pyridyloxobutylate) that binds with DNA, resulting in pyridyloxobutyl (POB)-DNA adducts. Certain POB-DNA adducts can break down under strong acidic conditions, releasing 4-hydroxy-1-(3-pyridyl)-1-butanone. The primary DNA adduct formed by PAH is benzo(a)pyrene diol epoxide. Researchers have found detectable levels of DNA adducts derived from PAHs and TSNA in the oral cells of smokers and non-smokers exposed to ETS.[43,44]

Biomarkers in Urine

Cotinine and 3-hydroxycotinine: Urine (100 μL) spiked with labeled internal standards will be enzymatically hydrolyzed at 37°C overnight in an orbital shaker to release the conjugated analytes. We will add 0.85 mL of cold acetone to each sample and refrigerate for over 10 minutes, followed by centrifugation at 4000 RPM for 2 minutes to eliminate precipitates of salts, protein, and exogenous enzymes. The top urine/acetone layer will then be transferred into a new vial and partially dried to remove acetone. A 10μL of the remaining supernatant urine will be directly injected into the LC-ECI-MS/MS system for simultaneous determination of total COT and 3 OH-COT.[45] Calibrants, solvent blank, matrix blank, and duplicate QC samples will be concurrently analyzed.

Markers of oxidative stress: ETS constituents undergo metabolic activation, forming diol-epoxides. These reactive compounds generate reactive oxygen species that can interact with DNA base pairs resulting in DNA damage.[46] Over 30 different types of base modifications have been identified, of which 8-hydroxy-2'-deoxyguanosine

For peer review only - http://bmjopen.bmj.com/site/about/guidelines.xhtml

(8OHdG) is widely recognized as a biomarker of ‘oxidatively damaged DNA.’[47] Researchers have measured levels of 8OHdG in population-based studies using different matrices like venous blood, urine, placenta, cord blood, and saliva to investigate the potential health effects caused by ETS exposure.[48-51] 8OHdG will be measured using the LC-ESI-MS/MS method.[45]

PAH metabolites: PAHs, known for their carcinogenic and mutagenic properties, are mainly formed as a result of incomplete combustion of tobacco and other organic materials.[53] PAH metabolites including 2-naphthol, 1-hydroxypyrene, and other relevant metabolites of 4-ring or less will be determined in urine by high-performance liquid chromatography and fluorescence (HPLC-FLD) detection previously published and cross-validated by LC-ESI-MS/MS technique.[53] The method LOD is <0.05 ng/mL and RSD is <5%. Creatinine will be measured and used for normalization of urine dilution.

4-(methylnitrosamino)-1-(3-pyridyl)-1-butanol (NNAL): Children exposed to ETS inhale TSNAs which are potent organ-specific carcinogens. The primary metabolite NNAL serves as an important biomarker of both exposure and effect and has been reported in pregnant women and children exposed to ETS.[54,55] We will employ a previously developed method for the simultaneous determination of total NNAL and NNN in urine by LC-ESI-MS/MS. This method has reported a LOD, accuracy, and precision of 0.05 pmol/mL, 110.9%, and 2.9%, respectively, for total NNAL.[56] These will be some of the first estimates of TSNA metabolites in children exposed to ETS from India.

Biomarkers in DBS

Hemoglobin (Hb) and Vitamin D: Iron deficiency in children increases the risk of ECC.[57] We plan to conduct Hb measurements using point-of-care devices (HemoCue® Hb 201 system) for immediate results that can be shared with physicians if necessary.

Vitamin D plays a crucial role in oral health. Inadequate vitamin D levels during periods of tooth development affect tooth calcification, predisposing enamel hypoplasia, and ECC.[58] 25 hydroxyvitamin D2 and 25 hydroxyvitamin D3 will be extracted from 6mm DBS, derivatized with 4-phenyl-1,2,4-triazoline-3,5-dione (PTAD) before analysis by LC-ESI-MS/MS method.[59]

Telomere length (TL): Telomeres are terminal DNA-protein structures that maintain the stability and integrity of chromosomes.[61] They are at risk of damage from reactive oxygen species produced by ETS components. Studies have demonstrated a dose-response relationship between prenatal ETS exposure and TL attrition.[61,62] Briefly, genomic DNA extraction will be performed using the QIAcube and QIAamp Mini Kit according to the manufacturer’s protocol. The average relative TL, represented by TL/single copy gene (T/S) ratio will be determined by quantitative-polymerase chain reaction (qPCR) using the absolute human TL quantification assay kit (ScienCell, CA, US) in conformance with minimum reporting recommendations of the Telomere Research Network, Tulane University School of Medicine, LA, US. Each sample will be run in duplicate, with a standard curve and two internal QC samples included on each plate.

For peer review only - http://bmjopen.bmj.com/site/about/guidelines.xhtml

Metal(loid)s analysis: A full spot which corresponds to approximately 60 µL of capillary blood will be excised with a ceramic blade into Teflon® vials. A blank spot will be cut from the same filter card to correct for background contamination. The weights of the digestion vials before and after will be documented to consider the precise mass of digested blood. Metal concentrations will be measured using QQQ-ICP-MS on a single run. Certified reference standard, Seronorm Serum L2 (Sero, Norway) will be analyzed once daily as a QC sample. Multiple isotopes of each metal will be monitored along with blanks, duplicates, and standard reference material to ensure method accuracy and precision.[63]

Fluoride concentration in water

Fluoride deficiency, especially widespread in socioeconomically disadvantaged areas, is a well-established risk factor for ECC. Therefore, we will gather information on toothpaste usage and collect water samples for fluoride analysis. Water samples will be collected from a subset of 50 homes and 25 common drinking water sources in the community. Concentration of fluoride in the water will be measured using a fluoride ion-selective electrode (Orion 9609BNWP, Thermo Scientific Inc., USA) connected to a bench-top analyzer. The data obtained will be recorded in ppm. Prior to each measurement, the electrode will be calibrated with four standard fluoride solutions: 1ppm, 10ppm, 50ppm, and 100ppm.

Quality Assurance/Quality Control

Each filter card and collection cup will be labeled with unique participant IDs and bar codes. To ensure a continuous cold chain, temperature-controlled Credo ProMed™ portable cooler bags will be used for collecting and transporting samples from field sites to the laboratory. Upon their arrival at the HAPIN BioMonitoring lab at SRIHER, the samples will undergo a cross-check against their tagged IDs and the chain of custody forms to verify accuracy before storing in deep freezers for long-term bio-archival. Trained personnel will record the quantity, quality, and condition of the DBS. Any smudged or double-dropped spots will be documented. If a spot is too small to accommodate a single small hole punch (3 mm), it will be deemed inadequate. Conversely, spots that can accommodate a single large hole punch (6 mm) will be considered suitable and those that fill the circle will be categorized as excellent.[64] Three to four aliquots of appropriate volumes will be prepared for saliva and urine to minimize multiple freeze-thaw cycles and facilitate long-term storage in a -80°C deep freezer. Field blanks and transport blanks will be employed to detect any potential contamination during sample collection, handling, and transport processes.

Data Management and Reporting

To maintain confidentiality, patients will be allocated unique identification numbers which will be used on all data collection forms and questionnaires. Data obtained will be secured/encrypted, computer data files will be password protected, and other study materials will be stored in locked file cabinets. We will collate the data sheets and create a final dataset in Excel. This will undergo thorough validation to ensure coherence and identify any potential errors.

For peer review only - http://bmjopen.bmj.com/site/about/guidelines.xhtml

Statistical Analysis

Baseline characteristics will be summarized using frequency and percentage for categorical variables and mean and SD for continuous variables with symmetric shape, otherwise median and interquartile range. The external validity in mean differences between children with and without ECC will be assessed using t-tests and for the difference in proportions $\chi 2$ tests of homogeneity will be used. To group similarly affected tooth surfaces, Ward's minimum variance method, a type of agglomerative hierarchical clustering will be applied. Each surface will start as its cluster, and similar clusters will then be successively merged to form a clustering hierarchy. The similarity among clusters will be evaluated using the Euclidean distance.[65] To analyze the associations of the timing of the ETS exposure (*in utero*, early postnatal, and current) and stages of ECC, generalized linear mixed models with a log-it link function will be employed. We will fit adjusted models for each outcome, accounting for potential confounders (i.e., maternal age, parental educational levels, household income, child age, gender, preterm birth/low birth weight, and yearly household income). To examine whether the prevalence of ECC increased with pack months of ETS exposure at home, a logistic regression model will be used. Quantile g-computation will be applied to estimate the associations of metal mixtures with dental caries using the R package qgcomp.[66] We will explore the use of advanced statistical methods to assess interrelationships between ETS biomarkers, oxidative stress, and ECC outcomes. We will also implement novel statistical methodology and machine learning algorithms to build epigenetic predictors of ETS exposure and ECC severity. Furthermore, we will utilize bioinformatic tools to map sites of epigenetic modifications between cases and controls. All statistical analyses will be performed using R version 4.2.0 [67] and bioinformatic packages will be downloaded from Bioconductor.[68]

Study Power

Sample size calculation was based on a previous study which reported the prevalence of ECC in the Indian population as 49.6%.[18] We estimated a sample size of 384 participants with an absolute precision of 5% and a 95% confidence interval. The CARES - HAPIN cohort consisting of 735 children has adequate statistical power to detect any association between ETS exposure and ECC.

Dissemination

The study findings will be published in peer-reviewed journals and disseminated through a national-level symposium involving researchers, subject experts, and healthcare providers. A summary of the results will also be provided to all participating researchers and patients after the final report is prepared. The quantitative exposure-response relationships derived from the study will have valuable implications for scientific and policy communities at large. Therefore, we will aim to make this data widely accessible while upholding participants' consent and confidentiality agreements.

Practical Lessons from CARES - HAPIN Pilot Study

For peer review only - http://bmjopen.bmj.com/site/about/guidelines.xhtml

A preliminary investigation was conducted with 21 children to evaluate the adequacy of the research procedures (i.e., clinical assessment, questionnaire administration, and biospecimen collection). Half of the children exhibited high levels of dental caries, with an average of 37.1% affected surfaces. This pilot study enabled us to establish procedures for analyzing COT, 3OH-COT, and 8OHdG in saliva as well as DNA extraction, purification, and sequencing in DBS. Moreover, it was observed that a majority of children and parents were willing to participate in this study.

We encountered several challenges throughout the process, which provided important lessons:

- It is crucial to ensure that the study instruments are not only translated into the relevant local language but also culturally appropriate for the population being studied. Understanding and addressing cultural barriers in research holds significance.
- In longitudinal cohort studies, participant tracking can be challenging, especially with migrant populations. Maintaining regular communication with participants and obtaining contact details of individuals who are familiar with their whereabouts can prove beneficial. In our study, we found that obtaining the contact details of the child's mother was notably advantageous.
- Research staff must be adaptable and willing to work beyond the regular hours. Also, evaluations at primary health care centers may not always be suitable, necessitating the establishment of alternatives such as anganwadis (pre-schools) and/or home visits.
- Special consideration should be given to biological specimen collection in remote or rural environments, ensuring strict adherence to SOPs for sample integrity. Careful handling and storage of biological samples are paramount to gather extensive information. Regular quality assurance and QC measures should also be implemented.

DISCUSSION

ECC is an insidious adversary, affecting millions of children worldwide. If the study's hypothesis is supported, it will help to elucidate the potential biological pathways through which ETS exposure impacts dental caries, impelling the development of more targeted ECC interventions and therapies. Additionally, to our knowledge, this will be one of the first studies to demonstrate how ETS exposure can influence epigenetic programming with potential downstream consequences for developing dental caries later in life.

The strength of our approach includes leveraging the existing high-quality data and resources from the HAPIN cohort, which will enable us to cost-effectively recapitulate information on the prenatal and early postnatal environmental milieu. It has established comprehensive survey data on ETS exposure during pregnancy (maternal report only) and childhood (child and maternal report); a wealth of covariate data to rigorously adjust for confounders and precision variables across analyses; and a field network system to undertake robust ECC assessment. The HAPIN cohort has also created a databank with bioassay results (urinary COT, 3OHCOT, 8OHdG, and PAHs metabolites) and a biorepository, thereby, providing a resource to investigate modifiable stressors, that impact the child's trajectory of dental caries. Furthermore, it is sufficiently powered to analyze outcomes related to

For peer review only - http://bmjopen.bmj.com/site/about/guidelines.xhtml

harmful environmental exposures. This serves as a groundwork for in-depth analysis of the critical window or 'timing' of ETS exposure in relation to ECC occurrence. The research team is composed of experts who have carried out seminal work in all the relevant research domains on which the study is based, including environmental health and epidemiology, human biomonitoring, epigenetics, and biostatistics. This ensures that the study maintains both high standards in design and execution, while also remaining practical and pragmatic throughout. We recognize that the successful implementation of the study hinges on a meticulous collection, storage, and analysis of biospecimens. The experience gained from conducting the CARES - HAPIN pilot study has proved to be valuable in refining the main research endeavor. As a result, minor adjustments have been made to improve how information is presented to parents and children, as well as streamline the biospecimen collection. Our work will provide insight into how simultaneous environmental exposures at critical time points interact among themselves, with individual characteristics (e.g., genetics), and epigenetics - a key component to reduce the ECC prevalence. This will highlight opportunities for policy recommendations.

References

1. Prüss-Üstün A. Preventing disease through healthy environments. Towards an estimate of the environmental burden of disease. WHO, Geneva 2006.
2. Barker DJP. The origins of the developmental origins theory. J Intern Med. 2007;261(5):412–417.
3. Selevan SG, Kimmel CA, Mendola P. Identifying critical windows of exposure for children's health. Environ Health Perspect. 2000;108 Suppl 3:451-5.
4. Wild CP. The exposome: from concept to utility. Int J Epidemiol. 2012;41(1):24-32.
5. Wild CP. Complementing the genome with an "exposome": the outstanding challenge of environmental exposure measurement in molecular epidemiology. Cancer Epidemiol Biomarkers Prev. 2005;14(8):1847–1850.
6. Hanioka T, Ojima M, Tanaka K, Yamamoto M. Does secondhand smoke affect the development of dental caries in children? A systematic review. Int J Environ Res Public Health. 2011;8:1503-19.
7. González-Valero L, Montiel-Company JM, Bellot-Arcís C, Almerich- Torres T, Iranzo-Cortés JE, Almerich-Silla JM. Association between passive tobacco exposure and caries in children and adolescents. A systematic review and meta-analysis. PLoS ONE. 2018;13:e0202497.
8. Zhong Y, Tang Q, Tan B, Huang R. Correlation Between Maternal Smoking During Pregnancy and Dental Caries in Children: A Systematic Review and Meta-Analysis. Front Oral Health. 2021;2:673449.
9. Uthayakumar T, Bennett JX, Cartas HL, Brunet M, Vo KL, Kroon J. Passive Smoking and Oral Health of Infants, Preschoolers, and Children: A Systematic Review. Nicotine Tob Res. 2023;25(10):1625-1632.
10. Davis E, Bakulski KM, Goodrich JM, Peterson KE, Marazita ML, Foxman B. Low levels of salivary metals, oral microbiome composition and dental decay. Sci Rep. 2020;10(1):14640.
11. Global oral health status report: towards universal health coverage for oral health by 2030. Geneva: World Health Organization; 2022.

For peer review only

12. U.S. Department of Health and Human Services. National Call to Action to Promote Oral Health. Rockville, MD: U.S. Department of Health and Human Services, Public Health Service, National Institutes of Health, National Institute of Dental and Craniofacial Research. NIH Publication No. 03-5303, Spring 2003.
13. Pitts NB, Zero DT, Marsh PD, Ekstrand K, Weintraub JA, Ramos-Gomez F, Tagami J, Twetman S, Tsakos G, Ismail A. Dental caries. Nat Rev Dis Primers. 2017;3:17030.
14. Pitts NB, Baez RJ, Diaz-Guillory C, Donly KJ, Feldens CA, McGrath C, et al. Early Childhood Caries: IAPD Bangkok Declaration. J Dent Child (Chic). 2019;86(2):72.
15. Watt RG. Social determinants of oral health inequalities: implications for action. Community Dent Oral Epidemiol. 2012;40 Suppl 2:44-8.
16. Wen PYF, Chen MX, Zhong YJ, Dong QQ, Wong HM. Global Burden and Inequality of Dental Caries, 1990 to 2019. J Dent Res. 2022;101(4):392-399.
17. Tinanoff N, Baez RJ, Diaz Guillory C, Donly KJ, Feldens CA, McGrath C, et al. Early childhood caries epidemiology, aetiology, risk assessment, societal burden, management, education, and policy: Global perspective. Int J Paediatr Dent. 2019;29(3):238-248.
18. Ganesh A, Muthu MS, Mohan A, Kirubakaran R. Prevalence of Early Childhood Caries in India – A Systematic Review. Indian J Pediatr. 2018; 86(3):276-86.
19. International Agency for Research on Cancer. Tobacco smoke and involuntary smoking. IARC Monogr Eval Carcinog Risks Hum. 2004;83:1–1438.
20. Surgeon General. The health consequences of smoking-50 years of progress; A report of the Surgeon General. Department of Health and Human Services. Public Health Service. US Office of the Surgeon General. Rockville 2014. Available: http://www.surgeongeneral.gov/library/reports/50-years-of- progress/full-report.pdf. Accessed on 23 October 2023.
21. Öberg, M, Woodward A, Jaakkola, MS, Peruga, A, Prüss-Üstün, A. Global estimate of the burden of disease from second-hand smoke. World Health Organization. 2010.
22. Behbod B, Sharma M, Baxi R, Roseby R, Webster P. Family and carer smoking control programmes for reducing children's exposure to environmental tobacco smoke. Cochrane Database Syst Rev. 2018;1(1):CD001746.
23. Global Adult Tobacco Survey (GATS-2) India 2016–17. Available: https://ntcp.mohfw.gov.in/assSHS/document/surveys-reports-publications/Global-Adult-Tobacco-Survey-Second-Round-India-2016-2017.pdf. Accessed on 23 October 2023.
24. Chowdhury IG, Bromage TG. Effects of fetal exposure to nicotine on dental development of the laboratory rat. Anat Rec. 2000;258(4):397-405.
25. Strauss RS. Environmental tobacco smoke and serum vitamin C levels in children. Pediatrics. 2001;107:540-2.
26. Avşar A, Darka O, Bodrumlu EH, Bek Y. Evaluation of the relationship between passive smoking and salivary electrolytes, protein, secretory IgA, sialic acid and amylase in young children. Arch Oral Biol. 2009;54:457-63.

For peer review only - http://bmjopen.bmj.com/site/about/guidelines.xhtml

27. Reese SE, Zhao S, Wu MC, Joubert BR, Parr CL, Håberg SE, et al. DNA Methylation Score as a Biomarker in Newborns for Sustained Maternal Smoking during Pregnancy. Environ Health Perspect. 2017;125(4):760-766.
28. Clasen T, Checkley W, Peel JL, Balakrishnan K, McCracken JP, Rosa G,et al. Design and Rationale of the HAPIN Study: A Multicountry Randomized Controlled Trial to Assess the Effect of Liquefied Petroleum Gas Stove and Continuous Fuel Distribution. Environ Health Perspect. 2020;128(4):47008.
29. Pitts N, Ekstrand K. International Caries Detection and Assessment System (ICDAS) and its International Caries Classification and Management System (ICCMS)—methods for staging of the caries process and enabling dentists to manage caries. Community Dent Oral Epidemiol. 2013;41:e41–e52.
30. Barr DB, Puttaswamy N, Jaacks LM, Steenland K, Rajkumar S, Gupton S, et al. Design and Rationale of the Biomarker Center of the Household Air Pollution Intervention Network (HAPIN) Trial. Environ Health Perspect. 2020;128(4):47010.
31. Rose JE, Levin ED, Benowitz N. Saliva nicotine as an index of plasma levels in nicotine skin patch users. Ther Drug Monit. 1993;15:431–35.
32. Jarvis MJ, Primatesta P, Erens B, Feyerabend C, Bryant A. Measuring nicotine intake in population surveys: Comparability of saliva cotinine and plasma cotinine estimates. Nicotine Tob Res. 2003;5:349–55.
33. McDade TW, Williams S, Snodgrass JJ. What a drop can do: dried blood spots as a minimally invasive method for integrating biomarkers into population-based research. Demography. 2007;44(4):899-925.
34. Barr DB, Kannan K, Cui Y, Merrill L, Petrick LM, Meeker JD, et al. The use of dried blood spots for characterizing children's exposure to organic environmental chemicals. Environ Res. 2021;195:110796.
35. Yoshida A, Suzuki N, Nakano Y, Kawada M, Oho T, Koga T. Development of a 5' nuclease-based real-time PCR assay for quantitative detection of cariogenic dental pathogens Streptococcus mutans and Streptococcus sobrinus. J Clin Microbiol. 2003;41(9):4438-41.
36. Sharma P, Sane N, Anand SD, Marimutthu P, Benegal V. Assessment of cotinine in urine and saliva of smokers, passive smokers, and nonsmokers: Method validation using liquid chromatography and mass spectrometry. Indian J Psychiatry. 2019;61(3):270-276.
37. Gatzke-Kopp LM, Riis JL, Ahmadi H, Piccerillo HL, Granger DA, Blair CB, Thomas EA. Environmental tobacco smoke exposure is associated with increased levels of metals in children's saliva. J Expo Sci Environ Epidemiol. 2023;33(6):903-910.
38. Chen Y, Ji P, Ma G, Song Z, Tang BQ, Li T. Simultaneous determination of cellular adenosine nucleotides, malondialdehyde, and uric acid using HPLC. Biomed Chromatogr. 2021;35(10):e5156.
39. Aryee M, Jaffe A, Corrada-Bravo H, Ladd-Acosta C, Feinberg A, Hansen K, et al. Minfi: a flexible and comprehensive Bioconductor package for the analysis of Infinium DNA methylation microarrays. Bioinformatics. 2014;30(10):1363–1369.
40. Triche TJ, Weisenberger DJ, Van Den Berg D, Laird PW, Siegmund KD. Low-level processing of Illumina Infinium DNA Methylation BeadArrays. Nucleic Acids Res. 2013;41(7):e90–e90.

For peer review only - http://bmjopen.bmj.com/site/about/guidelines.xhtml

41. Stepanov I, Muzic J, Le CT, Sebero E, Villalta P, Ma B, Jensen J, Hatsukami D, Hecht SS. Analysis of 4-hydroxy-1-(3-pyridyl)-1-butanone (HPB)-releasing DNA adducts in human exfoliated oral mucosa cells by liquid chromatography-electrospray ionization-tandem mass spectrometry. Chem Res Toxicol. 2013;26(1):37-45.
42. Chuang CY, Tung JN, Su MC, Wu BC, Hsin CH, Chen YJ, Yeh KT, Lee H, Cheng YW. BPDE-like DNA adduct level in oral tissue may act as a risk biomarker of oral cancer. Arch Oral Biol. 2013;58(1):102-9.
43. Paiano V, Maertens L, Guidolin V, Yang J, Balbo S, Hecht SS. Quantitative Liquid Chromatography-Nanoelectrospray Ionization-High-Resolution Tandem Mass Spectrometry Analysis of Acrolein-DNA Adducts and Etheno-DNA Adducts in Oral Cells from Cigarette Smokers and Nonsmokers. Chem Res Toxicol. 2020;33(8):2197-2207.
44. Chen KM, Sun YW, Krebs NM, Sun D, Krzeminski J, Reinhart L, et al. Detection of DNA adducts derived from the tobacco carcinogens, benzo[a]pyrene and dibenzo[def,p]chrysene in human oral buccal cells. Carcinogenesis. 2022;43(8):746-753.
45. McGuffey JE, Wei B, Bernert JT, Morrow JC, Xia B, Wang L, Blount BC. Validation of a LC-MS/MS method for quantifying urinary nicotine, six nicotine metabolites and the minor tobacco alkaloids--anatabine and anabasine--in smokers' urine. PLoS One. 2014;9(7):e101816.
46. van den Hooven EH, Pierik FH, de Kluizenaar Y, Willemsen SP, Hofman A, van Ratingen SW, et al. Air pollution exposure during pregnancy, ultrasound measures of fetal growth, and adverse birth outcomes: a prospective cohort study. Environ Health Perspect. 2012;120(1):150-6.
47. Malmqvist E, Liew Z, Källén K, Rignell-Hydbom A, Rittner R, Rylander L, et al. Fetal growth and air pollution - A study on ultrasound and birth measures. Environ Res. 2017;152:73-80.
48. Iñiguez C, Esplugues A, Sunyer J, Basterrechea M, Fernández-Somoano A, Costa O, et al. Prenatal Exposure to NO2 and Ultrasound Measures of Fetal Growth in the Spanish INMA Cohort. Environ Health Perspect. 2016;124(2):235-42.
49. Aguilera I, Garcia-Esteban R, Iñiguez C, Nieuwenhuijsen MJ, Rodríguez A, Paez M, et al. Prenatal exposure to traffic-related air pollution and ultrasound measures of fetal growth in the INMA Sabadell cohort. Environ Health Perspect. 2010;118(5):705-11.
50. Zhao N, Qiu J, Ma S, Zhang Y, Lin X, Tang Z, et al. Effects of prenatal exposure to ambient air pollutant PM10 on ultrasound-measured fetal growth. Int J Epidemiol. 2018;47(4):1072-1081.
51. Risom L, Møller P, Loft S. Oxidative stress-induced DNA damage by particulate air pollution. Mutat Res. 2005;592(1-2):119-37.
52. IARC Working Group on the Evaluation of Carcinogenic Risks to Humans. Some non-heterocyclic polycyclic aromatic hydrocarbons and some related exposures. IARC Monogr Eval Carcinog Risks Hum. 2010;92:1-853.
53. Puttaswamy N, Saidam S, Rajendran G, Arumugam K, Gupton S, Williams EW, et al. Cross-validation of biomonitoring methods for polycyclic aromatic hydrocarbon metabolites in human urine: Results from the

For peer review only

formative phase of the Household Air Pollution Intervention Network (HAPIN) trial in India. J Chromatogr B Analyt Technol Biomed Life Sci. 2020;1154:122284.

54. Thomas JL, Guo H, Carmella SG, Balbo S, Han S, Davis A, et al. Metabolites of a tobacco-specific lung carcinogen in children exposed to secondhand or thirdhand tobacco smoke in their homes. Cancer Epidemiol Biomarkers Prev. 2011;20(6):1213-21.
55. Vardavas CI, Fthenou E, Patelarou E, Bagkeris E, Murphy S, Hecht SS, et al. Exposure to different sources of second-hand smoke during pregnancy and its effect on urinary cotinine and tobacco-specific nitrosamine (NNAL) concentrations. Tob Control. 2013;22(3):194-200.
56. Nikam SS, Gurjar M, Singhavi H, Patil A, Singh A, Villalta P, et al. Simultaneous analysis of urinary total 4-(methylnitrosamino)-1-(3-pyridyl)-1-butanol, N'-nitrosonornicotine, and cotinine by liquid chromatography-tandem mass-spectrometry. Sci Rep. 2021;11(1):20007.
57. Ji SQ, Han R, Huang PP, Wang SY, Lin H, Ma L. Iron deficiency and early childhood caries: a systematic review and meta-analysis. Chin Med J (Engl). 2021;134(23):2832-2837.
58. Li Z, Wei X, Shao Z, Liu H, Bai S. Correlation between vitamin D levels in serum and the risk of dental caries in children: a systematic review and meta-analysis. BMC Oral Health. 2023;23(1):768.
59. Eyles D, Anderson C, Ko P, Jones A, Thomas A, Burne T, et al. A sensitive LC/MS/MS assay of 25OH vitamin D3 and 25OH vitamin D2 in dried blood spots. Clin Chim Acta. 2009;403(1-2):145-51.
60. Blackburn EH. Structure and function of telomeres. Nature. 1991;350(6319):569-73.
61. Theall KP, McKasson S, Mabile E, Dunaway LF, Drury SS. Early hits and long-term consequences:tracking the lasting impact of prenatal smoke exposure on telomere length in children. Am J Public Health. 2013;103(Suppl 1):S133–35.
62. Lu L, Johnman C, McGlynn L, Mackay DF, Shiels PG, Pell JP. Association between exposure to second-hand smoke and telomere length: cross-sectional study of 1303 non-smokers. Int J Epidemiol. 2017;46(6):1978-1984.
63. Langer EK, Johnson KJ, Shafer MM, Gorski P, Overdier J, Musselman J, Ross JA. Characterization of the elemental composition of newborn blood spots using sector-field inductively coupled plasma-mass spectrometry. J Expo Sci Environ Epidemiol. 2011;21(4):355-64.
64. Williams SR, McDade TW. The use of dried blood spot sampling in the national social life, health, and aging project. J Gerontol B Psychol Sci Soc Sci. 2009;64(Suppl 1):i131-6.
65. Ward JH. Hierarchical grouping to optimize an objective function. J Am Stat Assoc. 1963;58(301):236-244.
66. Keil AP, Buckley JP, O'Brien KM, Ferguson KK, Zhao S, White AJ. 2020. A quantile-based g-computation approach to addressing the effects of exposure mixtures. Environ Health Perspect. 128(4):47004.
67. R Core Team. R: A language and environment for statistical computing [Internet]. Vienna, Austria: R Foundation for Statistical Computing; 2015.
68. Huber W, Carey VJ, Gentleman R, Anders S, Carlson M, Carvalho BS, et al. Orchestrating high-throughput genomic analysis with Bioconductor. Nat Methods. 2015;12(2):115–121.

For peer review only - http://bmjopen.bmj.com/site/about/guidelines.xhtml

Contributors - NP, SP and AC conceptualised the project and were involved in developing study methodology and funding acquisition. SP and NP drafted the manuscript. AC, DBB, SG and KB edited the manuscript contributing critical content expertise.

Funding - The CARES study is supported by the Fogarty International Center (D43TW010540) with participation from the National Institute of Dental and Craniofacial Research. The HAPIN trial is funded by the U.S. National Institutes of Health (cooperative agreement 1UM1HL134590) in collaboration with the Bill & Melinda Gates Foundation (OPP1131279).

Disclaimer - The funders had no role in study design, data collection and analysis, decision to publish, or preparation of the manuscript.

Competing interests - None declared.

Patient and public involvement Patients and/or the public will be involved in the dissemination plans of this research. Refer to the Methods section for further details.

Ethics approval - Institutional Ethic Committee (IEC-NI22/JUL/83/82), Sri Ramachandra Institute of Higher Education and Research.

Provenance and peer review - Not commissioned; externally peer reviewed.

Data sharing statement - The data utilised for this study are not publicly available but de-identified data may be available on request, subject to approval by the internal review board and under a formal data use agreement. Contact the corresponding author for more information.

For peer review only

Table 1: List of biomarkers and summary of analysis scheme planned in the CARES - HAPIN study

**Biomarker**	**Matrix**	**Subject**	**Informs about**	**Method**	**Reference**
COT, 3 OH-COT	Saliva and Urine	Child/Mother	Recent exposure to tobacco smoke	LC-ESI-MS/MS	Sharma et al. 2019[36]; McGuffey et al. 2014[45]
Metals	Saliva and DBS	Child	Exposure to ETS	QQQ-ICP-MS	Gatzke-Kopp et al. 2023[37]; Longer et al. 2011[63]
Malondialdehyde	Saliva	Child/Mother	Oxidative damage to membranes	HPLC-UV	Chen et al. 2021[38]
Uric acid	Saliva	Child/Mother	Cellular metabolism	HPLC-UV	Chen et al. 2021[38]
Total antioxidant capacity	Saliva	Child/Mother	Antioxidant capacity	FRAP	ELISA Kit
Reduced Glutathione	Saliva	Child/Mother	Antioxidant capacity	Immunoassay	ELISA Kit
Strep. Mutans *Lacto. Bacillus*	Saliva	Child/Mother	Cariogenic bacterial load	qRT-PCR	Yoshida et al. 2003[35]
DNA Methylation	Buccal cells	Child	Epigenetic modification	BeadArray	Triche et al. 2013[40]
DNA Adduct	Buccal cells	Child	DNA damage	LC-ESI-MS/MS	Stepanov et al. 2013[41]; Chuang et al. 2013[42]
8OHdG	Urine	Child	Damage to DNA base pair	LC-ESI-MS/MS	McGuffey et al. 2014[45]
PAH metabolites	Urine	Child	Exposure to PAHs	HPLC-FLD	Puttaswamy et al. 2020[52]
NNAL	Urine	Child	Exposure to carcinogenic TSNAs	LC-ESI-MS/MS	Nikam et al. 2021[56]
25 hydroxyvitamin D2 and 25 hydroxyvitamin D3	DBS	Child	Vitamin D reserve	LC-ESI-MS/MS	Eyles et al. 2009[59]
Telomere Length	Buccal cells	Child	Cellular ageing	qPCR assay kit	ScienCell, CA, US

Figure Legends:

Figure 1. Conceptual framework of the Caries Risk from exposure to Environmental tobacco Smoke - Household Air Pollution Intervention Network (CARES - HAPIN) study

For peer review only

Figure 2. General mechanism linking ETS exposure and ECC occurrence

For peer review only

For peer review only - http://bmjopen.bmj.com/site/about/guidelines.xhtml

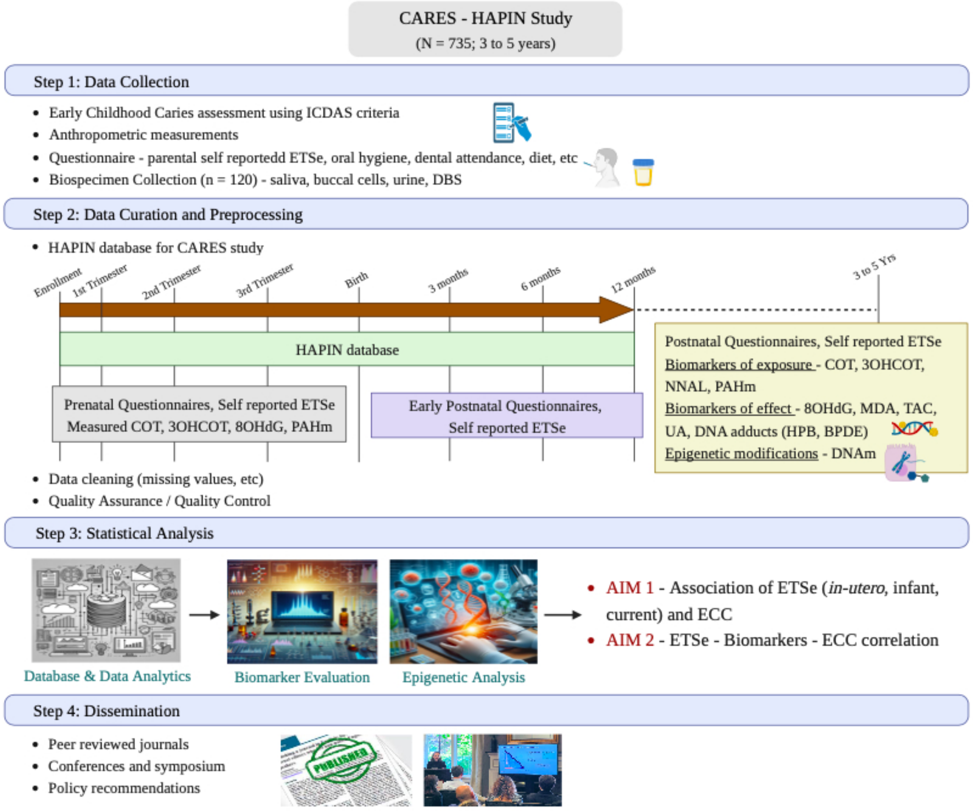

Conceptual framework of the Caries Risk from exposure to Environmental tobacco Smoke - Household Air Pollution Intervention Network (CARES - HAPIN) study

17x14mm (1200 x 1200 DPI)

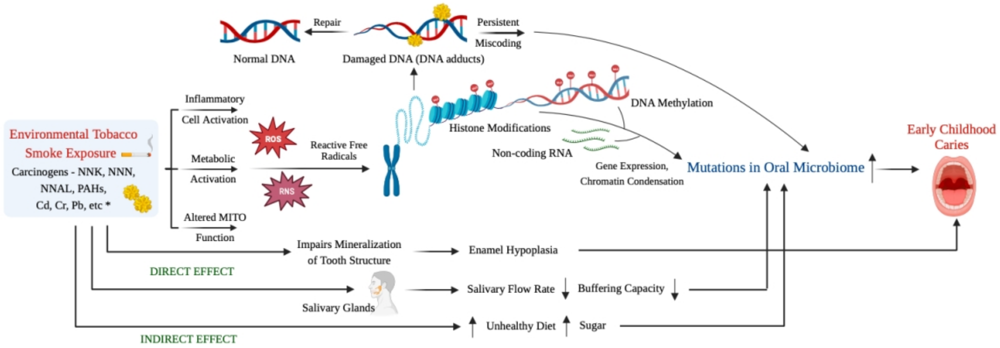

General mechanism linking ETS exposure and ECC occurrence

30x10mm (1200 x 1200 DPI)

BMJ Open

Exposure to Environmental Tobacco Smoke and Risk of Early Childhood Caries: Design and Rationale of the CARES - HAPIN Study

Journal:	*BMJ Open*
Manuscript ID	bmjopen-2024-083874.R1
Article Type:	Protocol
Date Submitted by the Author:	22-Mar-2024
Complete List of Authors:	Patil, Sneha; Sri Ramachandra Institute of Higher Education and Research (Deemed to be University), Department of Environmental Health Engineering, Faculty of Public Health; Dr. D.Y. Patil Dental College and Hospital, Dr. D.Y. Patil Vidyapeeth, Department of Pediatric and Preventive Dentistry<br>Puttaswamy, Naveen; Sri Ramachandra Institute of Higher Education and Research (Deemed to be University), Department of Environmental Health Engineering, Faculty of Public Health<br>Cardenas, Andres; Stanford School of Medicine, Stanford University, Department of Epidemiology and Population Health<br>Barr, Dana; Rollins School of Public Health, Emory University, Gangarosa Department of Environmental Health<br>Ghosh, Santu; St John's Medical College, Department of Biostatistics<br>Balakrishnan, Kalpana; Sri Ramachandra Institute of Higher Education and Research (Deemed to be University), Department of Environmental Health Engineering, Faculty of Public Health
**Primary Subject Heading**:	Dentistry and oral medicine
Secondary Subject Heading:	Public health, Epidemiology
Keywords:	PUBLIC HEALTH, Community child health < PAEDIATRICS, EPIDEMIOLOGIC STUDIES

SCHOLARONE™
Manuscripts

Protocol

Exposure to Environmental Tobacco Smoke and Risk of Early Childhood Caries: Design and Rationale of the CARES - HAPIN Study

Sneha S. Patil,[1,2] Naveen Puttaswamy,[1*] Andres Cardenas,[3] Dana Boyd Barr,[4] Santu Ghosh,[5] Kalpana Balakrishnan[1]

[1]Department of Environmental Health Engineering, Faculty of Public Health, Sri Ramachandra Institute of Higher Education and Research, Chennai, Tamil Nadu, India

[2] Department of Pediatric and Preventive Dentistry, Dr. D.Y. Patil Dental College and Hospital, Dr. D.Y. Patil Vidyapeeth, Sant-Tukaram Nagar, Pimpri, Pune, India

[3] Department of Epidemiology and Population Health, Stanford School of Medicine, Stanford University, Stanford, CA, USA

[4] Gangarosa Department of Environmental Health, Rollins School of Public Health, Emory University, Atlanta, GA, USA

[5] Department of Biostatistics, St. John's Medical College, Bengaluru, India

*Corresponding author

Naveen Puttaswamy

naveen@ehe.org.in

Contact no: +91 9003154028

For peer review only

ABSTRACT

Introduction: Prenatal and postnatal exposure to environmental tobacco smoke (ETS) has been linked with early childhood caries (ECC), but the specific molecular mechanisms and pathways remain largely unknown. The Caries Risk from exposure to Environmental tobacco Smoke (CARES) within the Household Air Pollution Intervention Network (HAPIN) study aims to establish the association between ETS and ECC by employing epidemiologic and novel biomarker-based approaches. Here we outline the overall design and rationale of the project.

Methods and analysis: We will leverage the infrastructure and data from the prospective birth cohort (HAPIN trial, India) to mount the CARES study. Children (n=735, aged: 3 to 5 years) will undergo ECC examination by a trained dentist using standard criteria and calibrated methods. Structured questionnaires will be used to gather information on sociodemographic variables, dietary habits, oral hygiene, oral health-related quality of life and current exposure to ETS. We will collect non-invasive or minimally invasive biospecimens (i.e., saliva, buccal cells, dried blood spots, and urine) from a subset of HAPIN children (n=120) to assess a battery of biomarkers indicative of exposure to ETS, early biological effect and epigenetic modifications. Both self-reported and objective measures of ETS exposure collected longitudinally at pre- and postnatal periods will be accessed from the HAPIN data. We will apply current science data techniques to assess the association and interrelationships between ETS, ECC, and multiple biomarkers.

Ethics and dissemination: Information gathered in this research will be published in peer-reviewed journals and summaries will be shared with the key stakeholders as well as patients and their parents/guardians involved in this study. Sri Ramachandra Institute of Higher Education and Research Ethics Board has approved the study protocol (IEC-NI22/JUL/83/82).

Keywords

Environmental tobacco smoke, early childhood caries, birth cohort, biomarkers, DNA methylation

Strengths and limitations of this study

- CARES-HAPIN dataset comprehensively captures the established risk factors for ECC, enabling us to accurately attribute the observed ECC to ETS exposure.
- Using longitudinal data on ETS exposure (*in-utero* and early childhood years) this research will identify the critical window of exposure to ETS associated with a greater risk of childhood caries development.
- First study to delve into the intricate biological pathways that link ETS exposure to ECC by applying multivariate statistics for in-depth analysis of high dimensional biomarker data.
- Furthermore, it seeks to unveil the profound impact of ETS exposure on epigenetic programming with potential implications for development of dental caries far into adulthood.
- CARES-HAPIN study limitation includes the extent to which findings are generalizable to other populations, since it predominantly comprises of children from a low socioeconomic status group.

For peer review only

For peer review only

For peer review only - http://bmjopen.bmj.com/site/about/guidelines.xhtml

INTRODUCTION

The World Health Organization estimates that approximately one-quarter of human diseases globally are attributable to environmental causes that can be obviated.[1] The first 1000 days of life (conception to 2 years), is a discrete window as it marks the beginning of rapid growth. Any disturbances due to environmental insults during this stage may have enduring impacts on health.[2,3] Environmental factors, inclusive of chemical contaminants, diet, lifestyle choices, social capital, stress, climate, and biological response to exposures collectively make up the exposome, the environmental complement of the genome.[4,5] The genetic code is largely static and non-modifiable, while the epigenome is modifiable and has garnered attention as a target for preventive strategies. This has led to an appreciation of the need to capture data from multiple omics layers using a data-driven approach to assess how environmental exposures influence individuals 'health. Emerging evidence indicates that exposure to environmental toxicants such as environmental tobacco smoke (ETS) and metal(loid)s can modify oral health.[6-10]

Oral diseases affect an estimated 3.5 billion people worldwide, with dental caries standing out as the most prevalent condition.[11] The stark inequalities, unparalleled burden, and shared risk factors with other non-communicable diseases, underline dental caries as a major public health problem.[11] Understanding the role of environmental pollutants is imperative for disease prevention as oral health is an integral aspect of general health.[11] This concern is particularly relevant for early childhood caries (ECC), as it is largely preventable and five times as common as asthma.[12,13] ECC is a dynamic, multifactorial disease, driven by biofilm and sugar, leading to phasic demineralization and remineralization of tooth structure.[14] In recent years, social determinants of health have gained prominence as crucial drivers of ECC.[15] Furthermore, it is closely linked to the 17 Sustainable Development Goals and strongly connected to six of the thirteen health targets in goal 3 - Good Health and Well-being.[16] Globally, more than 600 million children have untreated dental caries in primary teeth.[14] This not only causes pain and infection but the ramifications extend to negatively impact children's growth, neurodevelopment, school performance, and quality of life - all culminating in tremendous encumbrance on the family and society.[13,17] Notably, ECC is on the rise in racially and ethnically minoritized groups, as well as those living in low- and middle-income nations. Among Indian children, the prevalence of ECC varies between 42% to 63%.[18] This disparity arises from limited access to dental care services, financial barriers, scarcity of dental professionals, and lack of awareness about oral health.[13,17] For decades, researchers have meticulously studied the etiology of ECC and developed strategies to combat it. Despite this, its profound burden persists unabated. It may be prudent to explore environmental risk factors beyond the traditional factors in our quest to unravel this complex condition.

Mounting evidence evinces fetal and early-life exposure to ETS, even at low levels, increases the susceptibility of dental caries in children and adult life.[6-9] A recent meta-analysis has demonstrated that children residing with a smoker had significantly higher odds [prenatal - OR:1.57 (1.47–1.67); postnatal - OR:1.72 (1.45–2.05)] of presenting with dental caries.[7] ETS, an ubiquitous pollutant, also known as 'passive smoke 'or 'secondhand smoke ' refers to the mixture of smoke emitted from a burning tobacco product and exhaled by an active smoker.[19]

For peer review only - http://bmjopen.bmj.com/site/about/guidelines.xhtml

According to the 2014 US Surgeon General's report, there are numerous detrimental health outcomes related to ETS exposure including sudden infant death syndrome, preterm birth, impaired fetal growth, neurocognitive problems, cardiovascular diseases, and respiratory illness, all of which can persist into adulthood.[20] Recent systematic reviews and reports suggest that there is 'no safe level 'of ETS exposure.[19-22] In India, about 23 to 39% of the population is exposed to ETS, disproportionately affecting children and adolescent non-smokers.[23] The underlying mechanism linking ETS exposure to ECC pathogenesis remains poorly characterized, though several pathways including impaired salivary gland function, low levels of serum vitamin C, and enhanced streptococcus mutans proliferation are hypothesized to be involved.[24-26] Currently, questionnaires are commonly used for assessing pre- and postnatal ETS exposures; however, they are inadequate for measuring chemical exposures and suffer from misclassification, recall bias and reporting bias. Moreover, assessing exposure cross-sectionally at a given time point only provides a limited understanding of associations and fails to capture the complete range of lifelong exposures along with limited correlational interpretation. However, epigenetics, particularly DNA methylation (DNAm), offers a potential solution as it allows for the retention of environmental perturbations over time. For example, smoking-associated DNAm markers serve as biomarkers of smoke exposure, including the prenatal period.[27] Additionally, DNA adducts originating from tobacco specific nitrosamines (TSNAs) can also be used as markers of cumulative ETS exposure and early biological effects.

As yet, in low-resourced settings such as India where exposure magnitudes and ECC prevalence are among the highest, evidence for the role of ETS exposure on dental caries in childhood is lacking. Even less is known about the independent and combined impact of ETS exposure during fetal development and/or early childhood on ECC occurrence. In addition, there have been no published studies that have examined variation in DNAm associated with ETS exposure and ECC. To address this gap, we will adopt epidemiological and mechanistic approaches to test the association between pre- and postnatal ETS exposure and ECC in HAPIN children in India. This research aligns with the National Institute of Dental and Craniofacial Research and the Indian National Oral Health Program's mission to enhance oral health. Unpacking the complexity of biological pathways involved in environmental pollutants and ECC development and the challenges endured by children and their parents will add a major step towards personalized, precision environmental health and precision dental care.

Study Aims:

Using an integrated approach that includes ECC evaluation, ETS exposure measurements, and early biological markers of effect and susceptibility, we aim to: i) determine whether *in-utero*, infant, and current exposures to ETS (self-reported and measured) are associated with stages of ECC; ii) assess select biomarkers in saliva, buccal cells, dried blood spot (DBS) and urine indicative of exposure to ETS, oxidative stress, inflammation, and epigenetic modifications. A conceptual framework underlying this study is shown in fig.1

For peer review only - http://bmjopen.bmj.com/site/about/guidelines.xhtml

METHODS

Study Setting and Participants

Caries Risk from exposure to Environmental tobacco Smoke (CARES) is an ancillary study within the HAPIN cohort in India. The HAPIN trial (NCT02944682) was launched in 2017 across four diverse countries (India, Rwanda, Guatemala, and Peru) to assess the health benefits of clean cooking fuel intervention on birth outcomes, pneumonia, and child growth. A detailed description of the HAPIN population and protocol has been previously outlined.[28] In brief, between May 2018 and February 2020, 800 pregnant women were recruited from the Kallakurichi (KK) and Nagapattinam (NP) districts in Tamil Nadu, India. The study enrolled women between the ages of 18-35 years during antenatal visits at Primary Health Centres in the region, selecting those with a viable singleton pregnancy between 9 to < 20 weeks gestation, who were Tamil-speaking, and planning to deliver and receive prenatal care visits at local community clinics or hospitals. Assessments were conducted periodically during enrollment in early pregnancy, 24-28 weeks gestation, 32-36 weeks gestation, and infancy (3 months, 6 months, and 12 months). The study continues to follow up with mothers and their children, with data collected bi-annually. Of the initial 773 live births at the time of delivery, the HAPIN cohort is currently monitoring 735 children, aged between 3 to 5 years, who will be eligible for inclusion. Kalrayan Hills, KK district is home to 372 children who live at an altitude of 800 m above sea level, while the blocks of Keezhvelur, Kezhayur, Thalainayiru, and Vedaranyam in NP, a coastal district, have 363 children residing at elevations ranging from 10 to 50 m above sea level.

Ethical Aspects and Consent

CARES study protocol has been approved by the institutional review board of the participating institute. Agreements for sharing materials and data will be formulated based on the preferred standards of the institution and HAPIN guidelines. The conduct of this study will align with ethical principles outlined in the Belmont Report and follow Good Clinical Practice guidelines in line with the Indian Council of Medical Research.

Written informed consent will be obtained from the parent(s)/legal guardian(s) of all children. Consent protocol entails providing participants with comprehensive information regarding the aims, methods, potential risks, and benefits of participation. Parents will be informed that their child's participation is voluntary, and even if they choose not to, their child will still receive the necessary care in accordance with HAPIN guidelines. Moreover, parents will be reassured that appropriate measures are in place to safeguard their children's personal information and that any biospecimens collected will only be used for the specific study unless otherwise consented by them. They will be made aware that in the event an abnormality is detected during the analysis of samples, reports will be communicated to them, and further testing and medical interventions be advised as necessary.

Data Collection

Clinical Dental Examination

For peer review only - http://bmjopen.bmj.com/site/about/guidelines.xhtml

Dental caries assessment will be undertaken by visual examination using plain mouth mirrors, WHO CPI probe, and cotton rolls to remove debris and moisture when required. The diagnosis for each tooth surface will be based on modified International Caries Detection and Assessment (ICDAS) criteria; code 0 - indicates no evidence of caries, codes 1–2 initial lesions, codes 3–4 moderate, and 5–6 extensive caries lesions.[29] If a surface has multiple lesions, it will be coded according to the most severe presentation. To determine individual caries experience, the percentage of affected surfaces out of all examined surfaces will be calculated. The PUFA index will be employed to evaluate the consequences of untreated dental caries,[30] while the Plaque Index developed by Silness & Loe (1964) will be utilized to assess the plaque level. The intra-examiner reproducibility will be evaluated through two dental examinations conducted in 30 children at a 15-day interval by a trained and calibrated pediatric dentist. Additional clinical domains will encompass the evaluation of skeletal and occlusal characteristics, dental trauma, and developmental defects of the enamel.

Anthropometric Measurements

Children's height and weight will be measured by the trained surveyors. The US Centers for Disease Control and Prevention growth reference data will be used to compute body mass index, Z-scores and assign corresponding BMI categories (such as underweight, normal weight, overweight, and obese).

ETS Exposure Assessment

All pregnant women recruited in the HAPIN India site are non-smokers and 32% reported exposure to ETS in the baseline survey. Extant data on measures of cotinine (COT), 3-hydroxycotinine (3HC), and polycyclic aromatic hydrocarbons (PAH) during pregnancy and early childhood (up to 1 year of the child) will be accessed from the HAPIN database. At the time of the ECC assessment, we will capture the smoking status of family members by administering a short questionnaire to the parent(s)/legal guardian(s). Additionally, in a subset of the children, COT, 3HC, and PAH metabolites will be measured in saliva and urine to assess current exposure to ETS (details provided in the biomonitoring section). Both self-reported and objective measures of ETS exposure (*in-utero*, infant and current) will be used to assess the risk of caries development.

Questionnaire

Trained bilingual staff members will interview parents to collect information via detailed questionnaires on oral hygiene (initiation of brushing, frequency, parental supervision, use of fluoride toothpaste usage), diet (frequency of sweets consumption, eating before sleep), dental attendance (dental visit frequency and reasons), receipt of medications and child's oral health-related quality of life using the Early Childhood Oral Health Impact Scale (ECOHIS). The questionnaire has been designed by the research team referencing the American Academy of Pediatric Dentistry guidelines and pre-tested for clarity. Baseline data encompassing socioeconomic status, demographic details, household composition, pregnancy-related particulars, dietary diversity, and household

For peer review only - http://bmjopen.bmj.com/site/about/guidelines.xhtml

expenses will be obtained from the HAPIN research electronic data capture server. Information related to the child such as age, gender, type of delivery, only child, and feeding patterns will also be abstracted.

Biomonitoring

Biomarker analyses are central component of this study, drawing on existing biomarker research associated with ETS exposure and health. Biomarkers related to tobacco smoke exposure, inflammation, oxidative stress, and epigenetic changes have been chosen to inform mechanistic pathways to link ETS exposure and ECC occurrence; and also predict future dental caries risk (fig. 2). Standard protocols established for resource-limited field settings will be followed to ensure sample integrity when collecting biospecimens from children.[31]

Biological Matrix and Biomarker Selection

We will collect non-invasive or minimally invasive biospecimens including saliva, buccal cells, DBS, and urine from a subset (n=120) of the HAPIN children.

<u>*Saliva*</u>

Saliva is increasingly used as an alternative matrix for biomonitoring, particularly in children. Its collection is non-invasive, economical, easily accessible, and does not necessitate medical personnel in field settings with limited resources. Saliva provides recent physiological information about an individual through organic and inorganic components from the blood. Studies have shown a high correlation (r=0.94 to 0.99) in COT levels between saliva and plasma in both smokers and non-smokers.[32,33] Further, saliva is the preferred choice for assessing cariogenic bacteria counts and antioxidant status related to ECC. Saliva will be collected by passive drool from children ensuring no food or drink has been consumed 1 hour into the visit. They will be requested to sit still and silent in a comfortable position for 2-3 minutes. Saliva obtained in the first ten seconds will be discarded while that gathered thereafter within five minutes (approximately 1 to 2mL) will be pooled in a single collection vial. We will also collect saliva samples from mothers for correlation with COT, 3HC levels, and bacterial count assessments.

<u>*Buccal cells*</u>

Oral cell DNA is readily obtainable and constitutes a physiological domain impacted by ETS. ORAcollect pediatrics (OC-175, DNA Genotek, ON, Canada) DNA collection kits will be used to obtain buccal cells. The sponge tip will be positioned comfortably in the mouth and rubbed along both lower gums in a back-and-forth motion. After collection, the sponge tip will be placed into its corresponding tube containing the Oragene solution and gently mixed. Genomic DNA will be extracted with the prepIT•L2P kits from DNA Genotek and sample aliquots will be stored at −80°C until analysis.

<u>*Urine*</u>

Urine is a matrix of choice for environmental chemical metabolites that inform about biotransformation and internal exposure. Convenience or spot urine samples will be collected in sterile cups following ECC assessment. Urine will be aliquoted into cryo-chill vials and stored in deep freezers at -80°C until further analysis. A small aliquot will be used to measure creatinine using a semi-automatic biochemistry analyzer (Chem 7, Erba Mannheim, London UK)

For peer review only - http://bmjopen.bmj.com/site/about/guidelines.xhtml

through spectrophotometry. In addition, specific gravity will be measured with a urine refractometer PAL-10S (Atago Co. Ltd., Tokyo, Japan).

Dried blood spots (DBS)

We will collect routine blood samples as DBS on a five-spot Guthrie filter paper card (Whatman #903). This approach addresses the challenges associated with collecting, transporting, and storing venipuncture samples.[34] Also, DBS blood collection in children is minimally invasive, cost-effective, and requires low blood volume. In brief, the field staff will collect DBS sample from the children via finger prick after hand warming and arm massage to stimulate sufficient capillary blood flow for collection. The puncture site will be disinfected, and a sterile lancet will be used to puncture the ring or middle finger of the child's non-dominant hand. The first blood drop will be wiped away and subsequent blood drops will be applied to filter paper. Following sample collection, a sterile gauze pad will be pressed against the site until the bleeding stops. Samples will be air-dried for 24-48 hours and then placed in a biospecimen bag with desiccant and a humidity indicator card.[34,35]

Biomarker Measurements

The choice of biomarker under consideration in each matrix, the analytic technique, and its significance to the objectives of the CARES study is summarized in Table 1. The analytic protocol of each biomarker is provided in the sections below.

Biomarkers in Saliva

To evaluate the saliva flow rate, the collected saliva will be quantified in milliliters per minute, and pH levels checked within 30 minutes of collection. Salivary counts of Mutans streptococci and Lactobacilli will be determined using TaqMan PCR assay.[36] Total antioxidant capacity and reduced glutathione levels will be measured using the Ferric Reducing Antioxidant Power (FRAP) assay and by immunoassay technique, respectively.

Cotinine and 3-hydroxycotinine: The half-life of COT (~16h) is longer compared to nicotine (~2h) and is a predominant and stable metabolite of nicotine in body fluids. A simple, rapid, and sensitive method will be used to measure COT and 3HC simultaneously by the liquid chromatography-electrospray ionization-tandem mass spectrometry (LC-ESI-MS/MS) technique. An aliquot of saliva will be centrifuged in a cooling micro-centrifuge (Eppendorf 5430R) at 5000 rpm for 10 minutes. An equal volume of acetone will be added to the supernatant, with the final volume adjusted to 1mL using 0.1% formic acid in methanol solution. The resulting mixture will be vortex mixed and passed through Millex-HV syringe filters (4mm, 0.45μm) prior to analysis by LC-ESI-MS/MS. The limit of detection (LOD) for this method is 0.5 ng/mL, with a recovery of 99.6% and relative standard deviation (RSD) ranging from 9.2 to 9.5% for salivary COT in smoker's and non-smoker's samples.[37]

Metal(loid)s analysis: We will analyze trace metals in saliva and investigate their correlation with salivary COT to confirm if exposure to ETS contributes to the presence of metals in children. A recent study with 6-7-year-old children from the Family Life Project revealed positive correlations between salivary COT and Pb, Zn, and Cu, providing evidence for ETS as a source of trace metals.[38] Briefly, 0:5 mL of saliva will be weighted and digested

For peer review only - http://bmjopen.bmj.com/site/about/guidelines.xhtml

overnight in 2 mL of ultrapure concentrated HNO_3 acid. Subsequently, samples will be centrifuged at 15,000 rpm for 5 minutes at 4°C. The supernatant will be diluted at a 1:100 ratio with 1% nitric acid with indium as the internal standard and metal concentrations will then be measured on a single run of triple quadrupole inductively coupled plasma mass spectrometer (QQQ-ICP-MS, Agilent™-8900). Quality control (QC) measures will be employed to ensure accurate concentration estimates: procedural blanks, replicates, initial and continuous calibration verification. To assess the validity and reliability of measuring metals in the saliva of children, we will conduct a pilot analysis of paired samples (n=10) of saliva, venous blood, and DBS collected from healthy adult volunteers.

Malondialdehyde (MDA) and uric acid (UA): Measurement of MDA and UA is essential for studying mitochondrial functions as these components play a key role in energy metabolism and the production of reactive oxygen species. Saliva samples will be centrifuged at 5000 rpm for 10 min at 4°C and the supernatant will be used directly to measure MDA by colorimetric assay kits following manufacturer's procedure. UA will be quantified by Salimetrics UA assay kit (#1-3802) which has a wide assay range from 0.07 to 20 mg/dL and high sensitivity of 0.07 mg/dL LOD.

Biomarkers in Buccal Cells

DNA methylation (DNAm): Buccal cell DNA will be used to analyze DNAm with the Infinium Methylation EPIC v2.0 BeadChip which targets over 935,000 CpG sites. To ensure QC, replicates and randomization of samples across chips and plates will be employed. DNAm data will be processed using the minfi R package.[39] Samples not meeting certain criteria (e.g., duplicate samples, poor individual call rate <0.98, or genotyping/sex mismatch) will be excluded. Background correction, dye bias adjustment through the 'noob' method, and quantile normalization will be performed according to established procedures.[40]

DNA adducts: We will focus on quantifying DNA adducts formed by TSNAs and PAHs using the LC-ESI-MS/MS method as described elsewhere.[41,42] TSNAs such as 4-(N-nitrosomethylamino)-1-(3-pyridyl)-1-butanone (NNK) and N′-nitrosonornicotine (NNN) are found together in environments contaminated by tobacco smoke. The metabolic process involving α-hydroxylation at the 2'-position of NNN or the methyl group of NNK leads to the formation of a reactive intermediate (pyridyloxobutylate) that binds with DNA, resulting in pyridyloxobutyl (POB)-DNA adducts. Certain POB-DNA adducts can break down under strong acidic conditions, releasing 4-hydroxy-1-(3-pyridyl)-1-butanone. The primary DNA adduct formed by PAH is benzo(a)pyrene diol epoxide. Researchers have found detectable levels of DNA adducts derived from PAHs and TSNA in the oral cells of smokers and non-smokers exposed to ETS.[43,44]

Biomarkers in Urine

Cotinine and 3-hydroxycotinine: Urine (100 µL) spiked with labeled internal standards will be enzymatically hydrolyzed at 37°C overnight in an orbital shaker to release the conjugated analytes. We will add 0.85 mL of cold acetone to each sample and refrigerate for over 10 minutes, followed by centrifugation at 4000 rpm for 2 minutes to eliminate precipitates of salts, protein, and exogenous enzymes. The top urine/acetone layer will then be transferred into a new vial and partially dried to remove acetone. A 10µL of the remaining supernatant urine will

For peer review only - http://bmjopen.bmj.com/site/about/guidelines.xhtml

be directly injected into the LC-ECI-MS/MS system for simultaneous determination of total COT and 3HC.[45] Calibrants, solvent blank, matrix blank, and duplicate QC samples will be concurrently analyzed.

Markers of oxidative stress: ETS constituents undergo metabolic activation, forming diol-epoxides. These reactive compounds generate reactive oxygen species that can interact with DNA base pairs resulting in DNA damage.[46] Over 30 different types of base modifications have been identified, of which 8-hydroxy-2'-deoxyguanosine (8OHdG) is widely recognized as a biomarker of 'oxidatively damaged DNA.'[47] Researchers have measured levels of 8OHdG in population-based studies using different matrices like venous blood, urine, placenta, cord blood, and saliva to investigate the potential health effects caused by ETS exposure.[48-51] 8OHdG will be measured using the LC-ESI-MS/MS method.[45]

PAH metabolites: PAHs, known for their carcinogenic and mutagenic properties, are mainly formed as a result of incomplete combustion of tobacco and other organic materials.[52] PAH metabolites including 2-naphthol, 1-hydroxypyrene, and other relevant metabolites of 4-ring or less will be determined in urine by high-performance liquid chromatography and fluorescence (HPLC-FLD) detection previously published and cross-validated by LC-ESI-MS/MS technique.[53] The method LOD is <0.05 ng/mL and RSD is <5%. Creatinine will be measured and used for normalization of urine dilution.

4-(methylnitrosamino)-1-(3-pyridyl)-1-butanol (NNAL): Children exposed to ETS inhale TSNAs which are potent organ-specific carcinogens. The primary metabolite NNAL serves as an important biomarker of both exposure and effect and has been reported in pregnant women and children exposed to ETS.[54,55] We will employ a previously developed method for the simultaneous determination of total NNAL and NNN in urine by LC-ESI-MS/MS. This method has reported a LOD, accuracy, and precision of 0.05 pmol/mL, 110.9%, and 2.9%, respectively, for total NNAL.[56] These will be some of the first estimates of TSNA metabolites in children exposed to ETS from India.

Biomarkers in DBS

Hemoglobin (Hb) and Vitamin D: Iron deficiency in children increases the risk of ECC.[57] We plan to conduct Hb measurements using point-of-care devices (HemoCue® Hb 201 system) for immediate results that can be shared with physicians if necessary.

Vitamin D plays a crucial role in oral health. Inadequate vitamin D levels during periods of tooth development affect tooth calcification, predisposing enamel hypoplasia, and ECC.[58] 25 hydroxyvitamin D2 and 25 hydroxyvitamin D3 will be extracted from 6mm DBS, derivatized with 4-phenyl-1,2,4-triazoline-3,5-dione (PTAD) before analysis by LC-ESI-MS/MS method.[59]

Telomere length (TL): Telomeres are terminal DNA-protein structures that maintain the stability and integrity of chromosomes.[60] They are at risk of damage from reactive oxygen species produced by ETS components. Studies have demonstrated a dose-response relationship between prenatal ETS exposure and TL attrition.[61,62] Briefly, genomic DNA extraction will be performed using the QIAcube and QIAamp Mini Kit according to the manufacturer's protocol. The average relative TL, represented by TL/single copy gene (T/S) ratio will be

For peer review only

determined by quantitative-polymerase chain reaction (qPCR) using the absolute human TL quantification assay kit (ScienCell, CA, US) in conformance with minimum reporting recommendations of the Telomere Research Network, Tulane University School of Medicine, LA, US. Each sample will be run in duplicate, with a standard curve and two internal QC samples included on each plate.

Metal(loid)s analysis: Four spots of 3mm which corresponds to approximately 60 µL of capillary blood will be excised with a ceramic blade into Teflon® vials. A blank spot will be cut from the same filter card to correct for background contamination. The weights of the digestion vials before and after will be documented to consider the precise mass of digested blood. Metal concentrations will be measured using QQQ-ICP-MS on a single run. Certified reference standard, Seronorm Serum L2 (Sero, Norway) will be analyzed once daily as a QC sample. Multiple isotopes of each metal will be monitored along with blanks, duplicates, and standard reference material to ensure method accuracy and precision.[63]

Fluoride concentration in water

Fluoride deficiency, especially widespread in socioeconomically disadvantaged areas, is a well-established risk factor for ECC. Therefore, we will gather information on toothpaste usage and collect water samples for fluoride analysis. Water samples will be collected from a subset of 50 homes and 25 common drinking water sources in the community. Concentration of fluoride in the water will be measured using a fluoride ion-selective electrode (Orion 9609BNWP, Thermo Scientific Inc., USA) connected to a bench-top analyzer. The data obtained will be recorded in ppm. Prior to each measurement, the electrode will be calibrated with four standard fluoride solutions: 1ppm, 10ppm, 50ppm, and 100ppm.

Data Management and Reporting

To maintain confidentiality, patients will be allocated unique identification numbers which will be used on all data collection forms and questionnaires. Data obtained will be secured/encrypted, computer data files will be password protected, and other study materials will be stored in locked file cabinets. We will collate the data sheets and create a final dataset in Excel. This will undergo thorough validation to ensure coherence and identify any potential errors.

Quality Assurance/Quality Control

Each filter card and collection cup will be labeled with the unique participant IDs and bar codes. To ensure a continuous cold chain, temperature-controlled Credo ProMed™ portable cooler bags will be used for collecting and transporting samples from field sites to the laboratory. Upon their arrival at the HAPIN BioMonitoring lab at SRIHER, the samples will undergo a cross-check against their tagged IDs and the chain of custody forms to verify accuracy before storing in deep freezers for long-term bio-archival. Trained personnel will record the quantity, quality, and condition of the DBS. Any smudged or double-dropped spots will be documented. If a spot is too small to accommodate a single small hole punch (3 mm), it will be deemed inadequate. Conversely, spots that can accommodate a single large hole punch (6 mm) will be considered suitable and those that fill the circle will be

For peer review only - http://bmjopen.bmj.com/site/about/guidelines.xhtml

categorized as excellent.[64] Three to four aliquots of appropriate volumes will be prepared for saliva and urine to minimize multiple freeze-thaw cycles and facilitate long-term storage in a -80°C deep freezer. Field blanks and transport blanks will be employed to detect any potential contamination during sample collection, handling, and transport processes.

Statistical Analysis

Baseline characteristics will be summarized using frequency and percentage for categorical variables and mean and SD for continuous variables with symmetric shape, otherwise median and interquartile range. The external validity in mean differences between children with and without ECC will be assessed using t-tests and for the difference in proportions $\chi 2$ tests of homogeneity will be used. To group similarly affected tooth surfaces, Ward's minimum variance method, a type of agglomerative hierarchical clustering will be applied. Each surface will start as its cluster, and similar clusters will then be successively merged to form a clustering hierarchy. The similarity among clusters will be evaluated using the Euclidean distance.[65] To analyze the associations of the timing of the ETS exposure (in utero, early postnatal, and current) and stages of ECC, generalized linear mixed models with a log-it link function will be employed. We will fit adjusted models for each outcome, accounting for potential confounders (i.e., maternal age, parental educational levels, household income, child age, gender, preterm birth/low birth weight, and yearly household income). To examine whether the prevalence of ECC increased with pack months of ETS exposure at home, a logistic regression model will be used. Quantile g-computation will be applied to estimate the associations of metal mixtures with dental caries using the R package qgcomp.[66] We will explore the use of advanced statistical methods to assess interrelationships between ETS biomarkers, oxidative stress, and ECC outcomes. We will also implement novel statistical methodology and machine learning algorithms to build epigenetic predictors of ETS exposure and ECC severity. Furthermore, we will utilize bioinformatic tools to map sites of epigenetic modifications between cases and controls. All statistical analyses will be performed using R version 4.2.0[67] and bioinformatic packages will be downloaded from Bioconductor.[68]

Study Power

Sample size calculation was based on a previous study which reported the prevalence of ECC in the Indian population as 49.6%.[18] We estimated a sample size of 384 participants with an absolute precision of 5% and a 95% confidence interval. The CARES - HAPIN cohort consisting of 735 children has adequate statistical power to detect any association between ETS exposure and ECC.

Patient and Public Involvement

The study findings will be disseminated through a national-level symposium involving researchers, subject experts, and healthcare providers. A summary of the results will be provided to all participating researchers and patients after the final report is prepared. The quantitative exposure-response relationships derived from the study will have

For peer review only - http://bmjopen.bmj.com/site/about/guidelines.xhtml

valuable implications for scientific and policy communities at large. Therefore, we will aim to make this data widely accessible while upholding participants' consent and confidentiality agreements.

Practical Lessons from CARES - HAPIN Pilot Study

A preliminary investigation was conducted with 21 children to evaluate the adequacy of the research procedures (i.e., clinical assessment, questionnaire administration, and biospecimen collection). Half of the children exhibited high levels of dental caries, with an average of 37.1% affected surfaces. This pilot study enabled us to establish procedures for analyzing COT, 3HC, and 8OHdG in saliva as well as DNA extraction, purification, and sequencing in DBS. Moreover, it was observed that a majority of children and parents were willing to participate in this study.

We encountered several challenges throughout the process, which provided important lessons:

- It is crucial to ensure that the study instruments are not only translated into the relevant local language but also culturally appropriate for the population being studied. Understanding and addressing cultural barriers in research holds significance.
- In longitudinal cohort studies, participant tracking can be challenging, especially with migrant populations. Maintaining regular communication with participants and obtaining contact details of individuals who are familiar with their whereabouts can prove beneficial. In our study, we found that obtaining the contact details of the child's mother was notably advantageous.
- Research staff must be adaptable and willing to work beyond the regular hours. Also, evaluations at primary health care centers may not always be suitable, necessitating the establishment of alternatives such as anganwadis (pre-schools) and/or home visits.
- Special consideration should be given to biological specimen collection in remote or rural environments, ensuring strict adherence to SOPs for sample integrity. Careful handling and storage of biological samples are paramount to gather extensive information. Regular quality assurance and QC measures should also be implemented.

DISCUSSION

ECC is an insidious adversary, affecting millions of children worldwide. If the study's hypothesis is supported, it will help to elucidate the potential biological pathways through which ETS exposure impacts dental caries, impelling the development of more targeted ECC interventions and therapies. Additionally, to our knowledge, this will be one of the first studies to demonstrate how ETS exposure can influence epigenetic programming with potential downstream consequences for developing dental caries later in life.

The strength of our approach includes leveraging the existing high-quality data and resources from the HAPIN cohort, which will enable us to cost-effectively recapitulate information on the prenatal and early postnatal environmental milieu. It has an established comprehensive survey data on ETS exposure during pregnancy (maternal report only) and childhood (child and maternal report); a wealth of covariate data to rigorously adjust for confounders and precision variables across analyses; and a field network system to undertake robust ECC

For peer review only

assessment. The HAPIN cohort has also created a databank with bioassay results (urinary COT, 3HC, 8OHdG, and PAHs metabolites) and a biorepository, thereby, providing a resource to investigate modifiable stressors, that impact the child's trajectory of dental caries. Furthermore, it is sufficiently powered to analyze outcomes related to harmful environmental exposures. This serves as a groundwork for in-depth analysis of the critical window or 'timing' of ETS exposure in relation to ECC occurrence. The research team is composed of experts who have carried out seminal work in all the relevant research domains on which the study is based, including environmental health and epidemiology, human biomonitoring, epigenetics, and biostatistics. This ensures that the study maintains both high standards in design and execution, while also remaining practical and pragmatic throughout. We recognize that the successful implementation of the study hinges on a meticulous collection, storage, and analysis of biospecimens. The experience gained from conducting the CARES - HAPIN pilot study has proved to be valuable in refining the main research endeavor. As a result, minor adjustments have been made to improve how information is presented to parents and children, as well as streamline the biospecimen collection. Our work will provide insight into how simultaneous environmental exposures at critical time points interact among themselves, with individual characteristics (e.g., genetics), and epigenetics - a key component to reduce the ECC prevalence. This will highlight opportunities for policy recommendations.

REFERENCES

1. Prüss-Üstün A. Preventing disease through healthy environments. Towards an estimate of the environmental burden of disease. WHO, Geneva 2006.
2. Barker DJP. The origins of the developmental origins theory. J Intern Med. 2007;261(5):412–417.
3. Selevan SG, Kimmel CA, Mendola P. Identifying critical windows of exposure for children's health. Environ Health Perspect. 2000;108 Suppl 3:451-5.
4. Wild CP. The exposome: from concept to utility. Int J Epidemiol. 2012;41(1):24-32.
5. Wild CP. Complementing the genome with an "exposome": the outstanding challenge of environmental exposure measurement in molecular epidemiology. Cancer Epidemiol Biomarkers Prev. 2005;14(8):1847–1850.
6. Hanioka T, Ojima M, Tanaka K, Yamamoto M. Does secondhand smoke affect the development of dental caries in children? A systematic review. Int J Environ Res Public Health. 2011;8:1503-19.
7. González-Valero L, Montiel-Company JM, Bellot-Arcís C, Almerich- Torres T, Iranzo-Cortés JE, Almerich-Silla JM. Association between passive tobacco exposure and caries in children and adolescents. A systematic review and meta-analysis. PLoS ONE. 2018;13:e0202497.
8. Zhong Y, Tang Q, Tan B, Huang R. Correlation Between Maternal Smoking During Pregnancy and Dental Caries in Children: A Systematic Review and Meta-Analysis. Front Oral Health. 2021;2:673449.
9. Uthayakumar T, Bennett JX, Cartas HL, Brunet M, Vo KL, Kroon J. Passive Smoking and Oral Health of Infants, Preschoolers, and Children: A Systematic Review. Nicotine Tob Res. 2023;25(10):1625-1632.

For peer review only - http://bmjopen.bmj.com/site/about/guidelines.xhtml

10. Davis E, Bakulski KM, Goodrich JM, Peterson KE, Marazita ML, Foxman B. Low levels of salivary metals, oral microbiome composition and dental decay. Sci Rep. 2020;10(1):14640.
11. Global oral health status report: towards universal health coverage for oral health by 2030. Geneva: World Health Organization; 2022.
12. U.S. Department of Health and Human Services. National Call to Action to Promote Oral Health. Rockville, MD: U.S. Department of Health and Human Services, Public Health Service, National Institutes of Health, National Institute of Dental and Craniofacial Research. NIH Publication No. 03-5303, Spring 2003.
13. Pitts NB, Zero DT, Marsh PD, Ekstrand K, Weintraub JA, Ramos-Gomez F, et al. Dental caries. Nat Rev Dis Primers. 2017;3:17030.
14. Pitts NB, Baez RJ, Diaz-Guillory C, Donly KJ, Feldens CA, McGrath C, et al. Early Childhood Caries: IAPD Bangkok Declaration. J Dent Child (Chic). 2019;86(2):72.
15. Watt RG. Social determinants of oral health inequalities: implications for action. Community Dent Oral Epidemiol. 2012;40 Suppl 2:44-8.
16. Wen PYF, Chen MX, Zhong YJ, Dong QQ, Wong HM. Global Burden and Inequality of Dental Caries, 1990 to 2019. J Dent Res. 2022;101(4):392-399.
17. Tinanoff N, Baez RJ, Diaz Guillory C, Donly KJ, Feldens CA, McGrath C, et al. Early childhood caries epidemiology, aetiology, risk assessment, societal burden, management, education, and policy: Global perspective. Int J Paediatr Dent. 2019;29(3):238-248.
18. Ganesh A, Muthu MS, Mohan A, Kirubakaran R. Prevalence of Early Childhood Caries in India – A Systematic Review. Indian J Pediatr. 2018; 86(3):276-86.
19. International Agency for Research on Cancer. Tobacco smoke and involuntary smoking. IARC Monogr Eval Carcinog Risks Hum. 2004;83:1–1438.
20. Surgeon General. The health consequences of smoking-50 years of progress; A report of the Surgeon General. Department of Health and Human Services. Public Health Service. US Office of the Surgeon General. Rockville 2014. Available: http://www.surgeongeneral.gov/library/reports/50-years-of- progress/full-report.pdf. Accessed on 23 October 2023.
21. Öberg, M, Woodward A, Jaakkola, MS, Peruga, A, Prüss-Üstün, A. Global estimate of the burden of disease from second-hand smoke. World Health Organization. 2010.
22. Behbod B, Sharma M, Baxi R, Roseby R, Webster P. Family and carer smoking control programmes for reducing children's exposure to environmental tobacco smoke. Cochrane Database Syst Rev. 2018;1(1):CD001746.
23. Global Adult Tobacco Survey (GATS-2) India 2016–17. Available: https://ntcp.mohfw.gov.in/assSHS/document/surveys-reports-publications/Global-Adult-Tobacco-Survey-Second-Round-India-2016-2017.pdf. Accessed on 23 October 2023.
24. Chowdhury IG, Bromage TG. Effects of fetal exposure to nicotine on dental development of the laboratory rat. Anat Rec. 2000;258(4):397-405.

For peer review only

25. Strauss RS. Environmental tobacco smoke and serum vitamin C levels in children. Pediatrics. 2001;107:540-2.
26. Avşar A, Darka O, Bodrumlu EH, Bek Y. Evaluation of the relationship between passive smoking and salivary electrolytes, protein, secretory IgA, sialic acid and amylase in young children. Arch Oral Biol. 2009;54:457-63.
27. Reese SE, Zhao S, Wu MC, Joubert BR, Parr CL, Håberg SE, et al. DNA Methylation Score as a Biomarker in Newborns for Sustained Maternal Smoking during Pregnancy. Environ Health Perspect. 2017;125(4):760-766.
28. Clasen T, Checkley W, Peel JL, Balakrishnan K, McCracken JP, Rosa G,et al. Design and Rationale of the HAPIN Study: A Multicountry Randomized Controlled Trial to Assess the Effect of Liquefied Petroleum Gas Stove and Continuous Fuel Distribution. Environ Health Perspect. 2020;128(4):47008.
29. Pitts N, Ekstrand K. International Caries Detection and Assessment System (ICDAS) and its International Caries Classification and Management System (ICCMS)—methods for staging of the caries process and enabling dentists to manage caries. Community Dent Oral Epidemiol. 2013;41:e41–e52.
30. Monse B, Heinrich-Weltzien R, Benzian H, Holmgren C, van Palenstein Helderman W. PUFA -an index of clinical consequences of untreated dental caries. Community Dent Oral Epidemiol. 2010;38(1):77-82.
31. Barr DB, Puttaswamy N, Jaacks LM, Steenland K, Rajkumar S, Gupton S, et al. Design and Rationale of the Biomarker Center of the Household Air Pollution Intervention Network (HAPIN) Trial. Environ Health Perspect. 2020;128(4):47010.
32. Rose JE, Levin ED, Benowitz N. Saliva nicotine as an index of plasma levels in nicotine skin patch users. Ther Drug Monit. 1993;15:431–35.
33. Jarvis MJ, Primatesta P, Erens B, Feyerabend C, Bryant A. Measuring nicotine intake in population surveys: Comparability of saliva cotinine and plasma cotinine estimates. Nicotine Tob Res. 2003;5:349–55.
34. McDade TW, Williams S, Snodgrass JJ. What a drop can do: dried blood spots as a minimally invasive method for integrating biomarkers into population-based research. Demography. 2007;44(4):899-925.
35. Barr DB, Kannan K, Cui Y, Merrill L, Petrick LM, Meeker JD, et al. The use of dried blood spots for characterizing children's exposure to organic environmental chemicals. Environ Res. 2021;195:110796.
36. Yoshida A, Suzuki N, Nakano Y, Kawada M, Oho T, Koga T. Development of a 5' nuclease-based real-time PCR assay for quantitative detection of cariogenic dental pathogens Streptococcus mutans and Streptococcus sobrinus. J Clin Microbiol. 2003;41(9):4438-41.
37. Sharma P, Sane N, Anand SD, Marimutthu P, Benegal V. Assessment of cotinine in urine and saliva of smokers, passive smokers, and nonsmokers: Method validation using liquid chromatography and mass spectrometry. Indian J Psychiatry. 2019;61(3):270-276.
38. Gatzke-Kopp LM, Riis JL, Ahmadi H, Piccerillo HL, Granger DA, Blair CB, Thomas EA. Environmental tobacco smoke exposure is associated with increased levels of metals in children's saliva. J Expo Sci Environ Epidemiol. 2023;33(6):903-910.

For peer review only

39. Aryee M, Jaffe A, Corrada-Bravo H, Ladd-Acosta C, Feinberg A, Hansen K, et al. Minfi: a flexible and comprehensive Bioconductor package for the analysis of Infinium DNA methylation microarrays. Bioinformatics. 2014;30(10):1363–1369.
40. Triche TJ, Weisenberger DJ, Van Den Berg D, Laird PW, Siegmund KD. Low-level processing of Illumina Infinium DNA Methylation BeadArrays. Nucleic Acids Res. 2013;41(7):e90–e90.
41. Stepanov I, Muzic J, Le CT, Sebero E, Villalta P, Ma B, et al. Analysis of 4-hydroxy-1-(3-pyridyl)-1-butanone (HPB)-releasing DNA adducts in human exfoliated oral mucosa cells by liquid chromatography-electrospray ionization-tandem mass spectrometry. Chem Res Toxicol. 2013;26(1):37-45.
42. Chuang CY, Tung JN, Su MC, Wu BC, Hsin CH, Chen YJ, et al. BPDE-like DNA adduct level in oral tissue may act as a risk biomarker of oral cancer. Arch Oral Biol. 2013;58(1):102-9.
43. Paiano V, Maertens L, Guidolin V, Yang J, Balbo S, Hecht SS. Quantitative Liquid Chromatography-Nanoelectrospray Ionization-High-Resolution Tandem Mass Spectrometry Analysis of Acrolein-DNA Adducts and Etheno-DNA Adducts in Oral Cells from Cigarette Smokers and Nonsmokers. Chem Res Toxicol. 2020;33(8):2197-2207.
44. Chen KM, Sun YW, Krebs NM, Sun D, Krzeminski J, Reinhart L, et al. Detection of DNA adducts derived from the tobacco carcinogens, benzo[a]pyrene and dibenzo[def,p]chrysene in human oral buccal cells. Carcinogenesis. 2022;43(8):746-753.
45. McGuffey JE, Wei B, Bernert JT, Morrow JC, Xia B, Wang L, et al. Validation of a LC-MS/MS method for quantifying urinary nicotine, six nicotine metabolites and the minor tobacco alkaloids--anatabine and anabasine--in smokers' urine. PLoS One. 2014;9(7):e101816.
46. van den Hooven EH, Pierik FH, de Kluizenaar Y, Willemsen SP, Hofman A, van Ratingen SW, et al. Air pollution exposure during pregnancy, ultrasound measures of fetal growth, and adverse birth outcomes: a prospective cohort study. Environ Health Perspect. 2012;120(1):150-6.
47. Malmqvist E, Liew Z, Källén K, Rignell-Hydbom A, Rittner R, Rylander L, et al. Fetal growth and air pollution - A study on ultrasound and birth measures. Environ Res. 2017;152:73-80.
48. Iñiguez C, Esplugues A, Sunyer J, Basterrechea M, Fernández-Somoano A, Costa O, et al. Prenatal Exposure to NO2 and Ultrasound Measures of Fetal Growth in the Spanish INMA Cohort. Environ Health Perspect. 2016;124(2):235-42.
49. Aguilera I, Garcia-Esteban R, Iñiguez C, Nieuwenhuijsen MJ, Rodríguez A, Paez M, et al. Prenatal exposure to traffic-related air pollution and ultrasound measures of fetal growth in the INMA Sabadell cohort. Environ Health Perspect. 2010;118(5):705-11.
50. Zhao N, Qiu J, Ma S, Zhang Y, Lin X, Tang Z, et al. Effects of prenatal exposure to ambient air pollutant PM10 on ultrasound-measured fetal growth. Int J Epidemiol. 2018;47(4):1072-1081.
51. Risom L, Møller P, Loft S. Oxidative stress-induced DNA damage by particulate air pollution. Mutat Res. 2005;592(1-2):119-37.

For peer review only - http://bmjopen.bmj.com/site/about/guidelines.xhtml

52. IARC Working Group on the Evaluation of Carcinogenic Risks to Humans. Some non-heterocyclic polycyclic aromatic hydrocarbons and some related exposures. IARC Monogr Eval Carcinog Risks Hum. 2010;92:1-853.
53. Puttaswamy N, Saidam S, Rajendran G, Arumugam K, Gupton S, Williams EW, et al. Cross-validation of biomonitoring methods for polycyclic aromatic hydrocarbon metabolites in human urine: Results from the formative phase of the Household Air Pollution Intervention Network (HAPIN) trial in India. J Chromatogr B Analyt Technol Biomed Life Sci. 2020;1154:122284.
54. Thomas JL, Guo H, Carmella SG, Balbo S, Han S, Davis A, et al. Metabolites of a tobacco-specific lung carcinogen in children exposed to secondhand or thirdhand tobacco smoke in their homes. Cancer Epidemiol Biomarkers Prev. 2011;20(6):1213-21.
55. Vardavas CI, Fthenou E, Patelarou E, Bagkeris E, Murphy S, Hecht SS, et al. Exposure to different sources of second-hand smoke during pregnancy and its effect on urinary cotinine and tobacco-specific nitrosamine (NNAL) concentrations. Tob Control. 2013;22(3):194-200.
56. Nikam SS, Gurjar M, Singhavi H, Patil A, Singh A, Villalta P, et al. Simultaneous analysis of urinary total 4-(methylnitrosamino)-1-(3-pyridyl)-1-butanol, N'-nitrosonornicotine, and cotinine by liquid chromatography-tandem mass-spectrometry. Sci Rep. 2021;11(1):20007.
57. Ji SQ, Han R, Huang PP, Wang SY, Lin H, Ma L. Iron deficiency and early childhood caries: a systematic review and meta-analysis. Chin Med J (Engl). 2021;134(23):2832-2837.
58. Li Z, Wei X, Shao Z, Liu H, Bai S. Correlation between vitamin D levels in serum and the risk of dental caries in children: a systematic review and meta-analysis. BMC Oral Health. 2023;23(1):768.
59. Eyles D, Anderson C, Ko P, Jones A, Thomas A, Burne T, et al. A sensitive LC/MS/MS assay of 25OH vitamin D3 and 25OH vitamin D2 in dried blood spots. Clin Chim Acta. 2009;403(1-2):145-51.
60. Blackburn EH. Structure and function of telomeres. Nature. 1991;350(6319):569-73.
61. Theall KP, McKasson S, Mabile E, Dunaway LF, Drury SS. Early hits and long-term consequences:tracking the lasting impact of prenatal smoke exposure on telomere length in children. Am J Public Health. 2013;103(Suppl 1):S133–35.
62. Lu L, Johnman C, McGlynn L, Mackay DF, Shiels PG, Pell JP. Association between exposure to second-hand smoke and telomere length: cross-sectional study of 1303 non-smokers. Int J Epidemiol. 2017;46(6):1978-1984.
63. Langer EK, Johnson KJ, Shafer MM, Gorski P, Overdier J, Musselman J, et al. Characterization of the elemental composition of newborn blood spots using sector-field inductively coupled plasma-mass spectrometry. J Expo Sci Environ Epidemiol. 2011;21(4):355-64.
64. Williams SR, McDade TW. The use of dried blood spot sampling in the national social life, health, and aging project. J Gerontol B Psychol Sci Soc Sci. 2009;64(Suppl 1):i131-6.
65. Ward JH. Hierarchical grouping to optimize an objective function. J Am Stat Assoc. 1963;58(301):236-244.
66. Keil AP, Buckley JP, O'Brien KM, Ferguson KK, Zhao S, White AJ. A quantile-based g-computation approach to addressing the effects of exposure mixtures. Environ Health Perspect. 2020;128(4):47004.

For peer review only

67. R Core Team. R: A language and environment for statistical computing [Internet]. Vienna, Austria: R Foundation for Statistical Computing; 2015.

68. Huber W, Carey VJ, Gentleman R, Anders S, Carlson M, Carvalho BS, et al. Orchestrating high-throughput genomic analysis with Bioconductor. Nat Methods. 2015;12(2):115–121.

Contributors - NP, SP and AC conceptualised the project and were involved in developing study methodology and funding acquisition. SP and NP drafted the manuscript. AC, DBB, SG and KB edited the manuscript contributing critical content expertise.

Funding - The CARES study is supported by the Fogarty International Center (D43TW010540) with participation from the National Institute of Dental and Craniofacial Research. The HAPIN trial is funded by the U.S. National Institutes of Health (cooperative agreement 1UM1HL134590) in collaboration with the Bill & Melinda Gates Foundation (OPP1131279).

Competing interests - None declared.

Provenance and peer review - Not commissioned; externally peer reviewed.

Data sharing statement - The data utilised for this study are not publicly available but de-identified data may be available on request, subject to approval by the internal review board and under a formal data use agreement. Contact the corresponding author for more information.

For peer review only

Table 1: List of biomarkers and summary of analysis scheme planned in the CARES - HAPIN study

Biomarker	Matrix	Subject	Informs about	Method	Reference
COT, 3HC	Saliva and Urine	Child/Mother	Recent exposure to ETS	LC-ESI-MS/MS	Sharma et al. 2019;[37] McGuffey et al. 2014 [45]
Metals	Saliva and DBS	Child	Exposure to ETS	QQQ-ICP-MS	Gatzke-Kopp et al. 2023;[38] Langer et al. 2011 [63]
Malondialdehyde	Saliva	Child/Mother	Oxidative damage to membranes	HPLC-UV	MDA colorimetric assay kit
Uric acid	Saliva	Child/Mother	Cellular metabolism	HPLC-UV	Salimetrics UA assay kit
Total antioxidant capacity	Saliva	Child/Mother	Antioxidant capacity	FRAP	ELISA Kit
Reduced Glutathione	Saliva	Child/Mother	Antioxidant capacity	Immunoassay	ELISA Kit
Strep. Mutans Lacto. Bacillus	Saliva	Child/Mother	Cariogenic bacterial load	qRT-PCR	Yoshida et al. 2003 [36]
DNA Methylation	Buccal cells	Child	Epigenetic modification	BeadArray	Triche et al. 2013 [40]
DNA Adduct	Buccal cells	Child	DNA damage	LC-ESI-MS/MS	Stepanov et al. 2013;[41] Chuang et al. 2013 [42]
8OHdG	Urine	Child	Damage to DNA base pair	LC-ESI-MS/MS	McGuffey et al. 2014 [45]
PAH metabolites	Urine	Child	Exposure to PAHs	HPLC-FLD	Puttaswamy et al. 2020 [53]
NNAL	Urine	Child	Exposure to carcinogenic TSNAs	LC-ESI-MS/MS	Nikam et al. 2021 [56]
25 hydroxyvitamin D2 and 25 hydroxyvitamin D3	DBS	Child	Vitamin D reserve	LC-ESI-MS/MS	Eyles et al. 2009 [59]
Telomere Length	DBS	Child	Cellular ageing	qPCR assay kit	ScienCell, CA, US

For peer review only - http://bmjopen.bmj.com/site/about/guidelines.xhtml

Figure Legends:

Figure 1. Conceptual framework of the Caries Risk from exposure to Environmental tobacco Smoke - Household Air Pollution Intervention Network (CARES - HAPIN) study

Figure 2. General mechanism linking ETS exposure and ECC occurrence

For peer review only

For peer review only - http://bmjopen.bmj.com/site/about/guidelines.xhtml

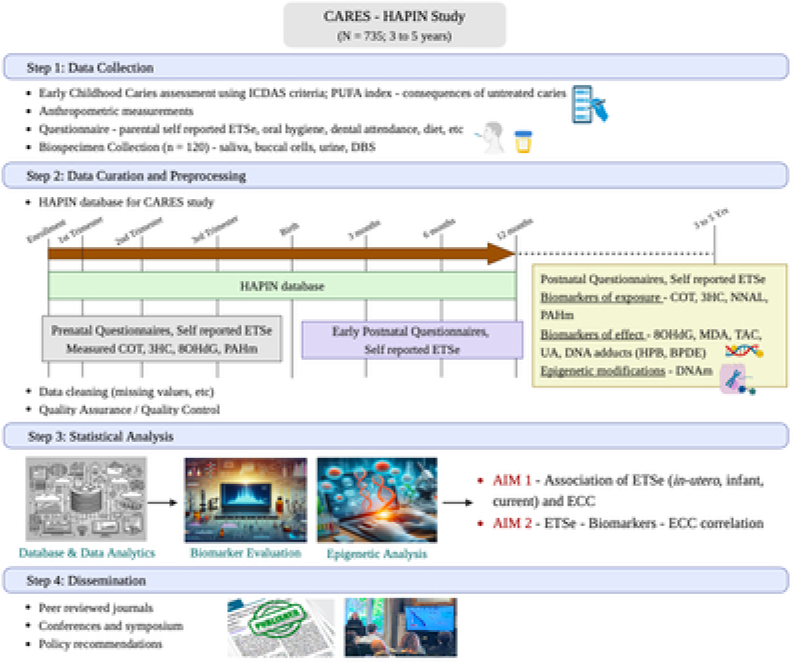

Conceptual framework of the Caries Risk from exposure to Environmental tobacco Smoke - Household Air Pollution Intervention Network (CARES - HAPIN) study

16x14mm (600 x 600 DPI)

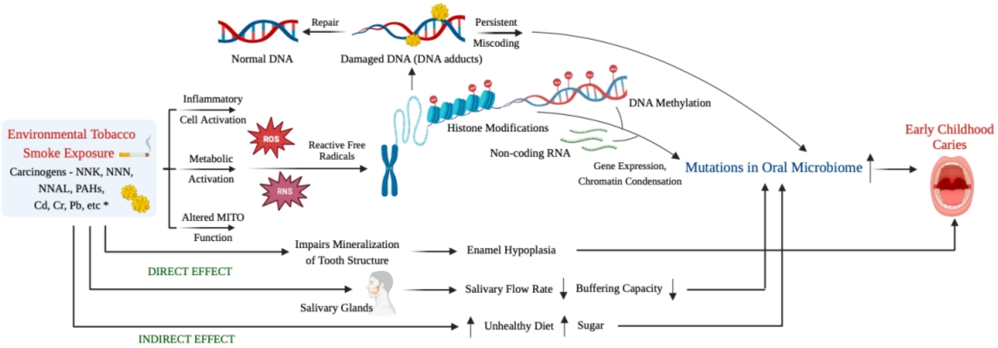

General mechanism linking ETS exposure and ECC occurrence

30x10mm (1200 x 1200 DPI)

BMJ Open

Protocol for CARES - HAPIN: An ambidirectional cohort study on exposure to environmental tobacco smoke and risk of early childhood caries

Journal:	*BMJ Open*
Manuscript ID	bmjopen-2024-083874.R2
Article Type:	Protocol
Date Submitted by the Author:	05-Apr-2024
Complete List of Authors:	Patil, Sneha; Sri Ramachandra Institute of Higher Education and Research (Deemed to be University), Department of Environmental Health Engineering, Faculty of Public Health; Dr. D.Y. Patil Dental College and Hospital, Dr. D.Y. Patil Vidyapeeth, Department of Pediatric and Preventive Dentistry<br>Puttaswamy, Naveen; Sri Ramachandra Institute of Higher Education and Research (Deemed to be University), Department of Environmental Health Engineering, Faculty of Public Health<br>Cardenas, Andres; Stanford School of Medicine, Stanford University, Department of Epidemiology and Population Health<br>Barr, Dana; Rollins School of Public Health, Emory University, Gangarosa Department of Environmental Health<br>Ghosh, Santu; St John's Medical College, Department of Biostatistics<br>Balakrishnan, Kalpana; Sri Ramachandra Institute of Higher Education and Research (Deemed to be University), Department of Environmental Health Engineering, Faculty of Public Health
**Primary Subject Heading**:	Dentistry and oral medicine
Secondary Subject Heading:	Public health, Epidemiology
Keywords:	PUBLIC HEALTH, Community child health < PAEDIATRICS, EPIDEMIOLOGIC STUDIES

SCHOLARONE™
Manuscripts

Protocol

Protocol for CARES - HAPIN: An ambidirectional cohort study on exposure to environmental tobacco smoke and risk of early childhood caries

Sneha S. Patil,[1,2] Naveen Puttaswamy,[1*] Andres Cardenas,[3] Dana Boyd Barr,[4] Santu Ghosh,[5] Kalpana Balakrishnan[1]

[1]Department of Environmental Health Engineering, Faculty of Public Health, Sri Ramachandra Institute of Higher Education and Research, Chennai, Tamil Nadu, India

[2] Department of Pediatric and Preventive Dentistry, Dr. D.Y. Patil Dental College and Hospital, Dr. D.Y. Patil Vidyapeeth, Sant-Tukaram Nagar, Pimpri, Pune, India

[3] Department of Epidemiology and Population Health, Stanford School of Medicine, Stanford University, Stanford, CA, USA

[4] Gangarosa Department of Environmental Health, Rollins School of Public Health, Emory University, Atlanta, GA, USA

[5] Department of Biostatistics, St. John's Medical College, Bengaluru, India

*Corresponding author

Naveen Puttaswamy

naveen@ehe.org.in

Contact no: +91 9003154028

For peer review only

ABSTRACT

Introduction: Pre- and postnatal exposure to environmental tobacco smoke (ETS) has been linked with early childhood caries (ECC), but the specific molecular mechanisms and pathways remain largely unknown. The Caries Risk from exposure to Environmental tobacco Smoke (CARES) within the Household Air Pollution Intervention Network (HAPIN) study aims to establish the association between ETS and ECC by employing epidemiologic and novel biomarker-based approaches. Here we outline the overall design and rationale of the project.

Methods and analysis: We will leverage the infrastructure and data from the HAPIN trial (India) to mount the CARES study. In this ambidirectional cohort study, children (n=735, aged: 3 to 5 years) will undergo ECC examination by a trained dentist using standard criteria and calibrated methods. Structured questionnaires will be used to gather information on sociodemographic variables, dietary habits, oral hygiene, oral health-related quality of life and current exposure to ETS. We will collect non-invasive or minimally invasive biospecimens (i.e., saliva, buccal cells, dried blood spots, and urine) from a subset of HAPIN children (n=120) to assess a battery of biomarkers indicative of exposure to ETS, early biological effect and epigenetic modifications. Both self-reported and objective measures of ETS exposure collected longitudinally at pre- and postnatal periods will be accessed from the HAPIN data. We will apply current science data techniques to assess the association and interrelationships between ETS, ECC, and multiple biomarkers.

Ethics and dissemination: Information gathered in this research will be published in peer-reviewed journals and summaries will be shared with the key stakeholders as well as patients and their parents/guardians involved in this study. Sri Ramachandra Institute of Higher Education and Research Ethics Board has approved the study protocol (IEC-NI22/JUL/83/82).

Keywords

Environmental tobacco smoke, early childhood caries, birth cohort, biomarkers, DNA methylation

Strengths and limitations of this study

- CARES-HAPIN dataset comprehensively captures the established risk factors for ECC, enabling us to accurately attribute the observed ECC to ETS exposure.
- Using longitudinal data on ETS exposure (*in-utero* and early childhood years) this research will identify the critical window of exposure to ETS associated with a greater risk of childhood caries development.
- First study to delve into the intricate biological pathways that link ETS exposure to ECC by applying multivariate statistics for in-depth analysis of high dimensional biomarker data.
- Limitation includes the extent to which findings are generalizable to other populations, since it predominantly comprises of children from a low socioeconomic status group.

For peer review only - http://bmjopen.bmj.com/site/about/guidelines.xhtml

INTRODUCTION

The World Health Organization estimates that approximately one-quarter of human diseases globally are attributable to environmental causes that can be obviated.[1] The first 1000 days of life (conception to 2 years), is a discrete window as it marks the beginning of rapid growth. Any disturbances due to environmental insults during this stage may have enduring impacts on health.[2,3] Environmental factors, inclusive of chemical contaminants, diet, lifestyle choices, social capital, stress, climate, and biological response to exposures collectively make up the exposome, the environmental complement of the genome.[4,5] The genetic code is largely static and non-modifiable, while the epigenome is modifiable and has garnered attention as a target for preventive strategies. This has led to an appreciation of the need to capture data from multiple omics layers using a data-driven approach to assess how environmental exposures influence individuals 'health. Emerging evidence indicates that exposure to environmental toxicants such as environmental tobacco smoke (ETS) and metal(loid)s can modify oral health.[6-10]

Oral diseases affect an estimated 3.5 billion people worldwide, with dental caries standing out as the most prevalent condition.[11] The stark inequalities, unparalleled burden, and shared risk factors with other non-communicable diseases, underline dental caries as a major public health problem.[11] Understanding the role of environmental pollutants is imperative for disease prevention as oral health is an integral aspect of general health.[11] This concern is particularly relevant for early childhood caries (ECC), as it is largely preventable and five times as common as asthma.[12,13] ECC is a dynamic, multifactorial disease, driven by biofilm and sugar, leading to phasic demineralization and remineralization of tooth structure.[14] In recent years, social determinants of health have gained prominence as crucial drivers of ECC.[15] Furthermore, it is closely linked to the 17 Sustainable Development Goals and strongly connected to six of the thirteen health targets in goal 3 - Good Health and Well-being.[16] Globally, more than 600 million children have untreated dental caries in primary teeth.[14] This not only causes pain and infection but the ramifications extend to negatively impact children's growth, neurodevelopment, school performance, and quality of life - all culminating in tremendous encumbrance on the family and society.[13,17] Notably, ECC is on the rise in racially and ethnically minoritized groups, as well as those living in low- and middle-income nations. Among Indian children, the prevalence of ECC varies between 42% to 63%.[18] This disparity arises from limited access to dental care services, financial barriers, scarcity of dental professionals, and lack of awareness about oral health.[13,17] For decades, researchers have meticulously studied the etiology of ECC and developed strategies to combat it. Despite this, its profound burden persists unabated. It may be prudent to explore environmental risk factors beyond the traditional factors in our quest to unravel this complex condition.

Mounting evidence evinces fetal and early-life exposure to ETS, even at low levels, increases the susceptibility of dental caries in children and adult life.[6-9] A recent meta-analysis has demonstrated that children residing with a smoker had significantly higher odds [prenatal - OR:1.57 (1.47–1.67); postnatal - OR:1.72 (1.45–2.05)] of presenting with dental caries.[7] ETS, an ubiquitous pollutant, also known as 'passive smoke 'or 'secondhand smoke ' refers to the mixture of smoke emitted from a burning tobacco product and exhaled by an active smoker.[19]

For peer review only - http://bmjopen.bmj.com/site/about/guidelines.xhtml

According to the 2014 US Surgeon General's report, there are numerous detrimental health outcomes related to ETS exposure including sudden infant death syndrome, preterm birth, impaired fetal growth, neurocognitive problems, cardiovascular diseases, and respiratory illness, all of which can persist into adulthood.[20] Recent systematic reviews and reports suggest that there is 'no safe level 'of ETS exposure.[19-22] In India, about 23 to 39% of the population is exposed to ETS, disproportionately affecting children and adolescent non-smokers.[23] The underlying mechanism linking ETS exposure to ECC pathogenesis remains poorly characterized, though several pathways including impaired salivary gland function, low levels of serum vitamin C, and enhanced *streptococcus mutans* proliferation are hypothesized to be involved.[24-26] Currently, questionnaires are commonly used for assessing pre- and postnatal ETS exposures; however, they are inadequate for measuring chemical exposures and suffer from misclassification, recall bias and reporting bias. Moreover, assessing exposure cross-sectionally at a given time point only provides a limited understanding of associations and fails to capture the complete range of lifelong exposures along with limited correlational interpretation. However, epigenetics, particularly DNA methylation (DNAm), offers a potential solution as it allows for the retention of environmental perturbations over time. For example, smoking-associated DNAm markers serve as biomarkers of smoke exposure, including the prenatal period.[27] Additionally, DNA adducts originating from tobacco specific nitrosamines (TSNAs) can also be used as markers of cumulative ETS exposure and early biological effects.

As yet, in low-resourced settings such as India where exposure magnitudes and ECC prevalence are among the highest, evidence for the role of ETS exposure on dental caries in childhood is lacking. Even less is known about the independent and combined impact of ETS exposure during fetal development and/or early childhood on ECC occurrence. In addition, there have been no published studies that have examined variation in DNAm associated with ETS exposure and ECC. To address this gap, we will adopt epidemiological and mechanistic approaches to test the association between pre- and postnatal ETS exposure and ECC in HAPIN children in India. This research aligns with the National Institute of Dental and Craniofacial Research and the Indian National Oral Health Program's mission to enhance oral health. Unpacking the complexity of biological pathways involved in environmental pollutants and ECC development and the challenges endured by children and their parents will add a major step towards personalized, precision environmental health and precision dental care.

Study Aims:

Using an integrated approach that includes ECC evaluation, ETS exposure measurements, and early biological markers of effect and susceptibility, we aim to: i) determine whether *in-utero*, infant, and current exposures to ETS (self-reported and measured) are associated with stages of ECC; ii) assess select biomarkers in saliva, buccal cells, dried blood spot (DBS) and urine indicative of exposure to ETS, oxidative stress, inflammation, and epigenetic modifications. A conceptual framework underlying this study is shown in fig.1

For peer review only - http://bmjopen.bmj.com/site/about/guidelines.xhtml

METHODS

Study Setting and Participants

Caries Risk from exposure to Environmental tobacco Smoke (CARES) is an ancillary study within the HAPIN cohort in India. The HAPIN trial (NCT02944682) was launched in 2017 across four diverse countries (India, Rwanda, Guatemala, and Peru) to assess the health benefits of clean cooking fuel intervention on birth outcomes, pneumonia, and child growth. A detailed description of the HAPIN population and protocol has been previously outlined.[28] In brief, between May 2018 and February 2020, 800 pregnant women were recruited from the Kallakurichi (KK) and Nagapattinam (NP) districts in Tamil Nadu, India. The study enrolled women between the ages of 18-35 years during antenatal visits at Primary Health Centres in the region, selecting those with a viable singleton pregnancy between 9 to < 20 weeks gestation, who were Tamil-speaking, and planning to deliver and receive prenatal care visits at local community clinics or hospitals. Assessments were conducted periodically during enrollment in early pregnancy, 24-28 weeks gestation, 32-36 weeks gestation, and infancy (3 months, 6 months, and 12 months). The study continues to follow up with mothers and their children, with data collected bi-annually. Of the initial 773 live births at the time of delivery, the HAPIN cohort is currently monitoring 735 children, aged between 3 to 5 years, who will be eligible for inclusion. Kalrayan Hills, KK district is home to 372 children who live at an altitude of 800 m above sea level, while the blocks of Keezhvelur, Kezhayur, Thalainayiru, and Vedaranyam in NP, a coastal district, have 363 children residing at elevations ranging from 10 to 50 m above sea level.

Data Collection

Clinical Dental Examination

Dental caries assessment will be undertaken by visual examination using plain mouth mirrors, WHO CPI probe, and cotton rolls to remove debris and moisture when required. The diagnosis for each tooth surface will be based on modified International Caries Detection and Assessment (ICDAS) criteria; code 0 - indicates no evidence of caries, codes 1–2 initial lesions, codes 3–4 moderate, and 5–6 extensive caries lesions.[29] If a surface has multiple lesions, it will be coded according to the most severe presentation. To determine individual caries experience, the percentage of affected surfaces out of all examined surfaces will be calculated. The PUFA index will be employed to evaluate the consequences of untreated dental caries,[30] while the Plaque Index developed by Silness & Loe (1964) will be utilized to assess the plaque level. The intra-examiner reproducibility will be evaluated through two dental examinations conducted in 30 children at a 15-day interval by a trained and calibrated pediatric dentist. Additional clinical domains will encompass the evaluation of skeletal and occlusal characteristics, dental trauma, and developmental defects of the enamel.

Anthropometric Measurements

For peer review only

Children's height and weight will be measured by the trained surveyors. The US Centers for Disease Control and Prevention growth reference data will be used to compute body mass index, Z-scores and assign corresponding BMI categories (such as underweight, normal weight, overweight, and obese).

ETS Exposure Assessment

All pregnant women recruited in the HAPIN India site are non-smokers and 32% reported exposure to ETS in the baseline survey. Extant data on measures of cotinine (COT), 3-hydroxycotinine (3HC), and polycyclic aromatic hydrocarbons (PAH) during pregnancy and early childhood (up to 1 year of the child) will be accessed from the HAPIN database. At the time of the ECC assessment, we will capture the smoking status of family members by administering a short questionnaire to the parent(s)/legal guardian(s). Additionally, in a subset of the children, COT, 3HC, and PAH metabolites will be measured in saliva and urine to assess current exposure to ETS (details provided in the biomonitoring section). Both self-reported and objective measures of ETS exposure (*in-utero*, infant and current) will be used to assess the risk of caries development.

Questionnaire

Trained bilingual staff members will interview parents to collect information via detailed questionnaires on oral hygiene (initiation of brushing, frequency, parental supervision, use of fluoride toothpaste usage), diet (frequency of sweets consumption, eating before sleep), dental attendance (dental visit frequency and reasons), receipt of medications and child's oral health-related quality of life using the Early Childhood Oral Health Impact Scale (ECOHIS). The questionnaire has been designed by the research team referencing the American Academy of Pediatric Dentistry guidelines and pre-tested for clarity. Baseline data encompassing socioeconomic status, demographic details, household composition, pregnancy-related particulars, dietary diversity, and household expenses will be obtained from the HAPIN research electronic data capture server. Information related to the child such as age, gender, type of delivery, only child, and feeding patterns will also be abstracted.

Biomonitoring

Biomarker analyses are central component of this study, drawing on existing biomarker research associated with ETS exposure and health. Biomarkers related to tobacco smoke exposure, inflammation, oxidative stress, and epigenetic changes have been chosen to inform mechanistic pathways to link ETS exposure and ECC occurrence; and also predict future dental caries risk (fig. 2). Standard protocols established for resource-limited field settings will be followed to ensure sample integrity when collecting biospecimens from children.[31]

Biological Matrix and Biomarker Selection

We will collect non-invasive or minimally invasive biospecimens including saliva, buccal cells, DBS, and urine from a subset (n=120) of the HAPIN children.

For peer review only

Saliva

Saliva is increasingly used as an alternative matrix for biomonitoring, particularly in children. Its collection is non-invasive, economical, easily accessible, and does not necessitate medical personnel in field settings with limited resources. Saliva provides recent physiological information about an individual through organic and inorganic components from the blood. Studies have shown a high correlation (r=0.94 to 0.99) in COT levels between saliva and plasma in both smokers and non-smokers.[32,33] Further, saliva is the preferred choice for assessing cariogenic bacteria counts and antioxidant status related to ECC. Saliva will be collected by passive drool from children ensuring no food or drink has been consumed 1 hour into the visit. They will be requested to sit still and silent in a comfortable position for 2-3 minutes. Saliva obtained in the first ten seconds will be discarded while that gathered thereafter within five minutes (approximately 1 to 2mL) will be pooled in a single collection vial. We will also collect saliva samples from mothers for correlation with COT, 3HC levels, and bacterial count assessments.

Buccal cells

Oral cell DNA is readily obtainable and constitutes a physiological domain impacted by ETS. ORAcollect pediatrics (OC-175, DNA Genotek, ON, Canada) DNA collection kits will be used to obtain buccal cells. The sponge tip will be positioned comfortably in the mouth and rubbed along both lower gums in a back-and-forth motion. After collection, the sponge tip will be placed into its corresponding tube containing the Oragene solution and gently mixed. Genomic DNA will be extracted with the prepIT•L2P kits from DNA Genotek and sample aliquots will be stored at −80°C until analysis.

Urine

Urine is a matrix of choice for environmental chemical metabolites that inform about biotransformation and internal exposure. Convenience or spot urine samples will be collected in sterile cups following ECC assessment. Urine will be aliquoted into cryo-chill vials and stored in deep freezers at -80°C until further analysis. A small aliquot will be used to measure creatinine using a semi-automatic biochemistry analyzer (Chem 7, Erba Mannheim, London UK) through spectrophotometry. In addition, specific gravity will be measured with a urine refractometer PAL-10S (Atago Co. Ltd., Tokyo, Japan).

Dried blood spots (DBS)

We will collect routine blood samples as DBS on a five-spot Guthrie filter paper card (Whatman #903). This approach addresses the challenges associated with collecting, transporting, and storing venipuncture samples.[34] Also, DBS blood collection in children is minimally invasive, cost-effective, and requires low blood volume. In brief, the field staff will collect DBS sample from the children via finger prick after hand warming and arm massage to stimulate sufficient capillary blood flow for collection. The puncture site will be disinfected, and a sterile lancet will be used to puncture the ring or middle finger of the child's non-dominant hand. The first blood drop will be wiped away and subsequent blood drops will be applied to filter paper. Following sample collection, a sterile gauze pad will be pressed against the site until the bleeding stops. Samples will be air-dried for 24-48 hours and then placed in a biospecimen bag with desiccant and a humidity indicator card.[34,35]

For peer review only - http://bmjopen.bmj.com/site/about/guidelines.xhtml

Biomarker Measurements

The choice of biomarker under consideration in each matrix, the analytic technique, and its significance to the objectives of the CARES study is summarized in Table 1. The analytic protocol of each biomarker is provided in the sections below.

Biomarkers in Saliva

To evaluate the saliva flow rate, the collected saliva will be quantified in milliliters per minute, and pH levels checked within 30 minutes of collection. Salivary counts of *Streptococcus Mutans* and *Lactobacilli* will be determined using TaqMan PCR assay.[36] Total antioxidant capacity and reduced glutathione levels will be measured using the Ferric Reducing Antioxidant Power (FRAP) assay and by immunoassay technique, respectively. *Cotinine and 3-hydroxycotinine:* The half-life of COT (~16h) is longer compared to nicotine (~2h) and is a predominant and stable metabolite of nicotine in body fluids. A simple, rapid, and sensitive method will be used to measure COT and 3HC simultaneously by the liquid chromatography-electrospray ionization-tandem mass spectrometry (LC-ESI-MS/MS) technique. An aliquot of saliva will be centrifuged in a cooling micro-centrifuge (Eppendorf 5430R) at 5000 rpm for 10 minutes. An equal volume of acetone will be added to the supernatant, with the final volume adjusted to 1mL using 0.1% formic acid in methanol solution. The resulting mixture will be vortex mixed and passed through Millex-HV syringe filters (4mm, 0.45μm) prior to analysis by LC-ESI-MS/MS. The limit of detection (LOD) for this method is 0.5 ng/mL, with a recovery of 99.6% and relative standard deviation (RSD) ranging from 9.2 to 9.5% for salivary COT in smoker's and non-smoker's samples.[37]

Metal(loid)s analysis: We will analyze trace metals in saliva and investigate their correlation with salivary COT to confirm if exposure to ETS contributes to the presence of metals in children. A recent study with 6-7-year-old children from the Family Life Project revealed positive correlations between salivary COT and Pb, Zn, and Cu, providing evidence for ETS as a source of trace metals.[38] Briefly, 0:5 mL of saliva will be weighted and digested overnight in 2 mL of ultrapure concentrated HNO_3 acid. Subsequently, samples will be centrifuged at 15,000 rpm for 5 minutes at 4°C. The supernatant will be diluted at a 1:100 ratio with 1% nitric acid with indium as the internal standard and metal concentrations will then be measured on a single run of triple quadrupole inductively coupled plasma mass spectrometer (QQQ-ICP-MS, Agilent™-8900). Quality control (QC) measures will be employed to ensure accurate concentration estimates: procedural blanks, replicates, initial and continuous calibration verification. To assess the validity and reliability of measuring metals in the saliva of children, we will conduct a pilot analysis of paired samples (n=10) of saliva, venous blood, and DBS collected from healthy adult volunteers. *Malondialdehyde (MDA) and uric acid (UA):* Measurement of MDA and UA is essential for studying mitochondrial functions as these components play a key role in energy metabolism and the production of reactive oxygen species. Saliva samples will be centrifuged at 5000 rpm for 10 min at 4°C and the supernatant will be used directly to measure MDA by colorimetric assay kits following manufacturer's procedure. UA will be quantified by Salimetrics

For peer review only - http://bmjopen.bmj.com/site/about/guidelines.xhtml

UA assay kit (#1-3802) which has a wide assay range from 0.07 to 20 mg/dL and high sensitivity of 0.07 mg/dL LOD.

Biomarkers in Buccal Cells

DNA methylation (DNAm): Buccal cell DNA will be used to analyze DNAm with the Infinium Methylation EPIC v2.0 BeadChip which targets over 935,000 CpG sites. To ensure QC, replicates and randomization of samples across chips and plates will be employed. DNAm data will be processed using the minfi R package.[39] Samples not meeting certain criteria (e.g., duplicate samples, poor individual call rate <0.98, or genotyping/sex mismatch) will be excluded. Background correction, dye bias adjustment through the 'noob' method, and quantile normalization will be performed according to established procedures.[40]

DNA adducts: We will focus on quantifying DNA adducts formed by TSNAs and PAHs using the LC-ESI-MS/MS method as described elsewhere.[41,42] TSNAs such as 4-(N-nitrosomethylamino)-1-(3-pyridyl)-1-butanone (NNK) and N′-nitrosonornicotine (NNN) are found together in environments contaminated by tobacco smoke. The metabolic process involving α-hydroxylation at the 2'-position of NNN or the methyl group of NNK leads to the formation of a reactive intermediate (pyridyloxobutylate) that binds with DNA, resulting in pyridyloxobutyl (POB)-DNA adducts. Certain POB-DNA adducts can break down under strong acidic conditions, releasing 4-hydroxy-1-(3-pyridyl)-1-butanone. The primary DNA adduct formed by PAH is benzo(a)pyrene diol epoxide. Researchers have found detectable levels of DNA adducts derived from PAHs and TSNA in the oral cells of smokers and non-smokers exposed to ETS.[43,44]

Biomarkers in Urine

Cotinine and 3-hydroxycotinine: Urine (100 μL) spiked with labeled internal standards will be enzymatically hydrolyzed at 37°C overnight in an orbital shaker to release the conjugated analytes. We will add 0.85 mL of cold acetone to each sample and refrigerate for over 10 minutes, followed by centrifugation at 4000 rpm for 2 minutes to eliminate precipitates of salts, protein, and exogenous enzymes. The top urine/acetone layer will then be transferred into a new vial and partially dried to remove acetone. A 10μL of the remaining supernatant urine will be directly injected into the LC-ECI-MS/MS system for simultaneous determination of total COT and 3HC.[45] Calibrants, solvent blank, matrix blank, and duplicate QC samples will be concurrently analyzed.

Markers of oxidative stress: ETS constituents undergo metabolic activation, forming diol-epoxides. These reactive compounds generate reactive oxygen species that can interact with DNA base pairs resulting in DNA damage.[46] Over 30 different types of base modifications have been identified, of which 8-hydroxy-2'-deoxyguanosine (8OHdG) is widely recognized as a biomarker of 'oxidatively damaged DNA.'[47] Researchers have measured levels of 8OHdG in population-based studies using different matrices like venous blood, urine, placenta, cord blood, and saliva to investigate the potential health effects caused by ETS exposure.[48-51] 8OHdG will be measured using the LC-ESI-MS/MS method.[45]

For peer review only - http://bmjopen.bmj.com/site/about/guidelines.xhtml

PAH metabolites: PAHs, known for their carcinogenic and mutagenic properties, are mainly formed as a result of incomplete combustion of tobacco and other organic materials.[52] PAH metabolites including 2-naphthol, 1-hydroxypyrene, and other relevant metabolites of 4-ring or less will be determined in urine by high-performance liquid chromatography and fluorescence (HPLC-FLD) detection previously published and cross-validated by LC-ESI-MS/MS technique.[53] The method LOD is <0.05 ng/mL and RSD is <5%. Creatinine will be measured and used for normalization of urine dilution.

4-(methylnitrosamino)-1-(3-pyridyl)-1-butanol (NNAL): Children exposed to ETS inhale TSNAs which are potent organ-specific carcinogens. The primary metabolite NNAL serves as an important biomarker of both exposure and effect and has been reported in pregnant women and children exposed to ETS.[54,55] We will employ a previously developed method for the simultaneous determination of total NNAL and NNN in urine by LC-ESI-MS/MS. This method has reported a LOD, accuracy, and precision of 0.05 pmol/mL, 110.9%, and 2.9%, respectively, for total NNAL.[56] These will be some of the first estimates of TSNA metabolites in children exposed to ETS from India.

Biomarkers in DBS

Hemoglobin (Hb) and Vitamin D: Iron deficiency in children increases the risk of ECC.[57] We plan to conduct Hb measurements using point-of-care devices (HemoCue® Hb 201 system) for immediate results that can be shared with physicians if necessary.

Vitamin D plays a crucial role in oral health. Inadequate vitamin D levels during periods of tooth development affect tooth calcification, predisposing enamel hypoplasia, and ECC.[58] 25 hydroxyvitamin D2 and 25 hydroxyvitamin D3 will be extracted from 6mm DBS, derivatized with 4-phenyl-1,2,4-triazoline-3,5-dione (PTAD) before analysis by LC-ESI-MS/MS method.[59]

Telomere length (TL): Telomeres are terminal DNA-protein structures that maintain the stability and integrity of chromosomes.[60] They are at risk of damage from reactive oxygen species produced by ETS components. Studies have demonstrated a dose-response relationship between prenatal ETS exposure and TL attrition.[61,62] Briefly, genomic DNA extraction will be performed using the QIAcube and QIAamp Mini Kit according to the manufacturer's protocol. The average relative TL, represented by TL/single copy gene (T/S) ratio will be determined by quantitative-polymerase chain reaction (qPCR) using the absolute human TL quantification assay kit (ScienCell, CA, US) in conformance with minimum reporting recommendations of the Telomere Research Network, Tulane University School of Medicine, LA, US. Each sample will be run in duplicate, with a standard curve and two internal QC samples included on each plate.

Metal(loid)s analysis: Four spots of 3mm which corresponds to approximately 60 µL of capillary blood will be excised with a ceramic blade into Teflon® vials. A blank spot will be cut from the same filter card to correct for background contamination. The weights of the digestion vials before and after will be documented to consider the precise mass of digested blood. Metal concentrations will be measured using QQQ-ICP-MS on a single run. Certified reference standard, Seronorm Serum L2 (Sero, Norway) will be analyzed once daily as a QC sample.

For peer review only - http://bmjopen.bmj.com/site/about/guidelines.xhtml

Multiple isotopes of each metal will be monitored along with blanks, duplicates, and standard reference material to ensure method accuracy and precision.[63]

Fluoride concentration in water

Fluoride deficiency, especially widespread in socioeconomically disadvantaged areas, is a well-established risk factor for ECC. Therefore, we will gather information on toothpaste usage and collect water samples for fluoride analysis. Water samples will be collected from a subset of 50 homes and 25 common drinking water sources in the community. Concentration of fluoride in the water will be measured using a fluoride ion-selective electrode (Orion 9609BNWP, Thermo Scientific Inc., USA) connected to a bench-top analyzer. The data obtained will be recorded in ppm. Prior to each measurement, the electrode will be calibrated with four standard fluoride solutions: 1ppm, 10ppm, 50ppm, and 100ppm.

Data Management and Reporting

To maintain confidentiality, patients will be allocated unique identification numbers which will be used on all data collection forms and questionnaires. Data obtained will be secured/encrypted, computer data files will be password protected, and other study materials will be stored in locked file cabinets. We will collate the data sheets and create a final dataset in Excel. This will undergo thorough validation to ensure coherence and identify any potential errors.

Quality Assurance/Quality Control

Each filter card and collection cup will be labeled with the unique participant IDs and bar codes. To ensure a continuous cold chain, temperature-controlled Credo ProMed™ portable cooler bags will be used for collecting and transporting samples from field sites to the laboratory. Upon their arrival at the HAPIN BioMonitoring lab at SRIHER, the samples will undergo a cross-check against their tagged IDs and the chain of custody forms to verify accuracy before storing in deep freezers for long-term bio-archival. Trained personnel will record the quantity, quality, and condition of the DBS. Any smudged or double-dropped spots will be documented. If a spot is too small to accommodate a single small hole punch (3 mm), it will be deemed inadequate. Conversely, spots that can accommodate a single large hole punch (6 mm) will be considered suitable and those that fill the circle will be categorized as excellent.[64] Three to four aliquots of appropriate volumes will be prepared for saliva and urine to minimize multiple freeze-thaw cycles and facilitate long-term storage in a -80°C deep freezer. Field blanks and transport blanks will be employed to detect any potential contamination during sample collection, handling, and transport processes.

Statistical Analysis

Baseline characteristics will be summarized using frequency and percentage for categorical variables and mean and SD for continuous variables with symmetric shape, otherwise median and interquartile range. The external validity in mean differences between children with and without ECC will be assessed using t-tests and for the difference in

For peer review only - http://bmjopen.bmj.com/site/about/guidelines.xhtml

proportions χ2 tests of homogeneity will be used. To group similarly affected tooth surfaces, Ward's minimum variance method, a type of agglomerative hierarchical clustering will be applied. Each surface will start as its cluster, and similar clusters will then be successively merged to form a clustering hierarchy. The similarity among clusters will be evaluated using the Euclidean distance.[65] To analyze the associations of the timing of the ETS exposure (*in utero*, early postnatal, and current) and stages of ECC, generalized linear mixed models with a log-it link function will be employed. We will fit adjusted models for each outcome, accounting for potential confounders (i.e., maternal age, parental educational levels, household income, child age, gender, preterm birth/low birth weight, and yearly household income). To examine whether the prevalence of ECC increased with pack months of ETS exposure at home, a logistic regression model will be used. Quantile g-computation will be applied to estimate the associations of metal mixtures with dental caries using the R package qgcomp.[66] We will explore the use of advanced statistical methods to assess interrelationships between ETS biomarkers, oxidative stress, and ECC outcomes. We will also implement novel statistical methodology and machine learning algorithms to build epigenetic predictors of ETS exposure and ECC severity. Furthermore, we will utilize bioinformatic tools to map sites of epigenetic modifications between cases and controls. All statistical analyses will be performed using R version 4.2.0 [67] and bioinformatic packages will be downloaded from Bioconductor.[68]

Study Power

Sample size calculation was based on a previous study which reported the prevalence of ECC in the Indian population as 49.6%.[18] We estimated a sample size of 384 participants with an absolute precision of 5% and a 95% confidence interval. The CARES - HAPIN cohort consisting of 735 children has adequate statistical power to detect any association between ETS exposure and ECC.

Patient and Public Involvement

Lay summaries of the results will be provided to patients, caregivers, and the wider community after the final report is prepared. Educational infographics illustrating the adverse effects of ETS exposure on pregnant women and fetal development will also be created for distribution at Primary Health Centres. The quantitative exposure-response relationship derived from the study will have valuable implications for both scientific inquiry and policy formulation. Therefore, we will aim to make this data widely accessible while upholding participants' consent and confidentiality agreements.

Ethics and Dissemination

CARES study protocol has been approved by the institutional review board of the participating institute (IEC-NI22/JUL/83/82). Agreements for sharing materials and data will be formulated based on the preferred standards of the institution and HAPIN guidelines. The conduct of this study will align with ethical principles outlined in the Belmont Report and follow Good Clinical Practice guidelines in line with the Indian Council of Medical Research.

For peer review only - http://bmjopen.bmj.com/site/about/guidelines.xhtml

Written informed consent will be obtained from the parent(s)/legal guardian(s) of all children. Consent protocol entails providing participants with comprehensive information regarding the aims, methods, potential risks, and benefits of participation. Parents will be informed that their child's participation is voluntary, and even if they choose not to, their child will still receive the necessary care in accordance with HAPIN guidelines. Moreover, parents will be reassured that appropriate measures are in place to safeguard their children's personal information and that any biospecimens collected will only be used for the specific study unless otherwise consented by them. They will be made aware that in the event an abnormality is detected during the analysis of samples, reports will be communicated to them, and further testing and medical interventions be advised as necessary.

The study findings will be published in peer-reviewed journals and disseminated through a national-level symposium involving researchers, subject experts, and healthcare providers.

Practical Lessons from CARES - HAPIN Pilot Study

A preliminary investigation was conducted with 21 children to evaluate the adequacy of the research procedures (i.e., clinical assessment, questionnaire administration, and biospecimen collection). Half of the children exhibited high levels of dental caries, with an average of 37.1% affected surfaces. This pilot study enabled us to establish procedures for analyzing COT, 3HC, and 8OHdG in saliva as well as DNA extraction, purification, and sequencing in DBS. Moreover, it was observed that a majority of children and parents were willing to participate in this study. We encountered several challenges throughout the process, which provided important lessons:

- It is crucial to ensure that the study instruments are not only translated into the relevant local language but also culturally appropriate for the population being studied. Understanding and addressing cultural barriers in research holds significance.
- In longitudinal cohort studies, participant tracking can be challenging, especially with migrant populations. Maintaining regular communication with participants and obtaining contact details of individuals who are familiar with their whereabouts can prove beneficial. In our study, we found that obtaining the contact details of the child's mother was notably advantageous.
- Research staff must be adaptable and willing to work beyond the regular hours. Also, evaluations at primary health care centers may not always be suitable, necessitating the establishment of alternatives such as anganwadis (pre-schools) and/or home visits.
- Special consideration should be given to biological specimen collection in remote or rural environments, ensuring strict adherence to SOPs for sample integrity. Careful handling and storage of biological samples are paramount to gather extensive information. Regular quality assurance and QC measures should also be implemented.

DISCUSSION

ECC is an insidious adversary, affecting millions of children worldwide. If the study's hypothesis is supported, it will help to elucidate the potential biological pathways through which ETS exposure impacts dental caries,

For peer review only - http://bmjopen.bmj.com/site/about/guidelines.xhtml

impelling the development of more targeted ECC interventions and therapies. Additionally, to our knowledge, this will be one of the first studies to demonstrate how ETS exposure can influence epigenetic programming with potential downstream consequences for developing dental caries later in life.

The strength of our approach includes leveraging the existing high-quality data and resources from the HAPIN cohort, which will enable us to cost-effectively recapitulate information on the prenatal and early postnatal environmental milieu. It has an established comprehensive survey data on ETS exposure during pregnancy (maternal report only) and childhood (child and maternal report); a wealth of covariate data to rigorously adjust for confounders and precision variables across analyses; and a field network system to undertake robust ECC assessment. The HAPIN cohort has also created a databank with bioassay results (urinary COT, 3HC, 8OHdG, and PAHs metabolites) and a biorepository, thereby, providing a resource to investigate modifiable stressors, that impact the child's trajectory of dental caries. Furthermore, it is sufficiently powered to analyze outcomes related to harmful environmental exposures. This serves as a groundwork for in-depth analysis of the critical window or 'timing' of ETS exposure in relation to ECC occurrence. The research team is composed of experts who have carried out seminal work in all the relevant research domains on which the study is based, including environmental health and epidemiology, human biomonitoring, epigenetics, and biostatistics. This ensures that the study maintains both high standards in design and execution, while also remaining practical and pragmatic throughout. We recognize that the successful implementation of the study hinges on a meticulous collection, storage, and analysis of biospecimens. The experience gained from conducting the CARES - HAPIN pilot study has proved to be valuable in refining the main research endeavor. As a result, minor adjustments have been made to improve how information is presented to parents and children, as well as streamline the biospecimen collection. Our work will provide insight into how simultaneous environmental exposures at critical time points interact among themselves, with individual characteristics (e.g., genetics), and epigenetics - a key component to reduce the ECC prevalence. This will highlight opportunities for policy recommendations.

REFERENCES

1. Prüss-Üstün A. Preventing disease through healthy environments. Towards an estimate of the environmental burden of disease. WHO, Geneva 2006.
2. Barker DJP. The origins of the developmental origins theory. J Intern Med. 2007;261(5):412–417.
3. Selevan SG, Kimmel CA, Mendola P. Identifying critical windows of exposure for children's health. Environ Health Perspect. 2000;108 Suppl 3:451-5.
4. Wild CP. The exposome: from concept to utility. Int J Epidemiol. 2012;41(1):24-32.
5. Wild CP. Complementing the genome with an "exposome": the outstanding challenge of environmental exposure measurement in molecular epidemiology. Cancer Epidemiol Biomarkers Prev. 2005;14(8):1847–1850.

For peer review only - http://bmjopen.bmj.com/site/about/guidelines.xhtml

6. Hanioka T, Ojima M, Tanaka K, Yamamoto M. Does secondhand smoke affect the development of dental caries in children? A systematic review. Int J Environ Res Public Health. 2011;8:1503-19.
7. González-Valero L, Montiel-Company JM, Bellot-Arcís C, Almerich- Torres T, Iranzo-Cortés JE, Almerich-Silla JM. Association between passive tobacco exposure and caries in children and adolescents. A systematic review and meta-analysis. PLoS ONE. 2018;13:e0202497.
8. Zhong Y, Tang Q, Tan B, Huang R. Correlation Between Maternal Smoking During Pregnancy and Dental Caries in Children: A Systematic Review and Meta-Analysis. Front Oral Health. 2021;2:673449.
9. Uthayakumar T, Bennett JX, Cartas HL, Brunet M, Vo KL, Kroon J. Passive Smoking and Oral Health of Infants, Preschoolers, and Children: A Systematic Review. Nicotine Tob Res. 2023;25(10):1625-1632.
10. Davis E, Bakulski KM, Goodrich JM, Peterson KE, Marazita ML, Foxman B. Low levels of salivary metals, oral microbiome composition and dental decay. Sci Rep. 2020;10(1):14640.
11. Global oral health status report: towards universal health coverage for oral health by 2030. Geneva: World Health Organization; 2022.
12. U.S. Department of Health and Human Services. National Call to Action to Promote Oral Health. Rockville, MD: U.S. Department of Health and Human Services, Public Health Service, National Institutes of Health, National Institute of Dental and Craniofacial Research. NIH Publication No. 03-5303, Spring 2003.
13. Pitts NB, Zero DT, Marsh PD, Ekstrand K, Weintraub JA, Ramos-Gomez F, et al. Dental caries. Nat Rev Dis Primers. 2017;3:17030.
14. Pitts NB, Baez RJ, Diaz-Guillory C, Donly KJ, Feldens CA, McGrath C, et al. Early Childhood Caries: IAPD Bangkok Declaration. J Dent Child (Chic). 2019;86(2):72.
15. Watt RG. Social determinants of oral health inequalities: implications for action. Community Dent Oral Epidemiol. 2012;40 Suppl 2:44-8.
16. Wen PYF, Chen MX, Zhong YJ, Dong QQ, Wong HM. Global Burden and Inequality of Dental Caries, 1990 to 2019. J Dent Res. 2022;101(4):392-399.
17. Tinanoff N, Baez RJ, Diaz Guillory C, Donly KJ, Feldens CA, McGrath C, et al. Early childhood caries epidemiology, aetiology, risk assessment, societal burden, management, education, and policy: Global perspective. Int J Paediatr Dent. 2019;29(3):238-248.
18. Ganesh A, Muthu MS, Mohan A, Kirubakaran R. Prevalence of Early Childhood Caries in India – A Systematic Review. Indian J Pediatr. 2018; 86(3):276-86.
19. International Agency for Research on Cancer. Tobacco smoke and involuntary smoking. IARC Monogr Eval Carcinog Risks Hum. 2004;83:1–1438.
20. Surgeon General. The health consequences of smoking-50 years of progress; A report of the Surgeon General. Department of Health and Human Services. Public Health Service. US Office of the Surgeon General. Rockville 2014. Available: http://www.surgeongeneral.gov/library/reports/50-years-of- progress/full-report.pdf. Accessed on 23 October 2023.

For peer review only

21. Öberg, M, Woodward A, Jaakkola, MS, Peruga, A, Prüss-Üstün, A. Global estimate of the burden of disease from second-hand smoke. World Health Organization. 2010.
22. Behbod B, Sharma M, Baxi R, Roseby R, Webster P. Family and carer smoking control programmes for reducing children's exposure to environmental tobacco smoke. Cochrane Database Syst Rev. 2018;1(1):CD001746.
23. Global Adult Tobacco Survey (GATS-2) India 2016–17. Available: https://ntcp.mohfw.gov.in/assSHS/document/surveys-reports-publications/Global-Adult-Tobacco-Survey-Second-Round-India-2016-2017.pdf. Accessed on 23 October 2023.
24. Chowdhury IG, Bromage TG. Effects of fetal exposure to nicotine on dental development of the laboratory rat. Anat Rec. 2000;258(4):397-405.
25. Strauss RS. Environmental tobacco smoke and serum vitamin C levels in children. Pediatrics. 2001;107:540-2.
26. Avşar A, Darka O, Bodrumlu EH, Bek Y. Evaluation of the relationship between passive smoking and salivary electrolytes, protein, secretory IgA, sialic acid and amylase in young children. Arch Oral Biol. 2009;54:457-63.
27. Reese SE, Zhao S, Wu MC, Joubert BR, Parr CL, Håberg SE, et al. DNA Methylation Score as a Biomarker in Newborns for Sustained Maternal Smoking during Pregnancy. Environ Health Perspect. 2017;125(4):760-766.
28. Clasen T, Checkley W, Peel JL, Balakrishnan K, McCracken JP, Rosa G,et al. Design and Rationale of the HAPIN Study: A Multicountry Randomized Controlled Trial to Assess the Effect of Liquefied Petroleum Gas Stove and Continuous Fuel Distribution. Environ Health Perspect. 2020;128(4):47008.
29. Pitts N, Ekstrand K. International Caries Detection and Assessment System (ICDAS) and its International Caries Classification and Management System (ICCMS)—methods for staging of the caries process and enabling dentists to manage caries. Community Dent Oral Epidemiol. 2013;41:e41–e52.
30. Monse B, Heinrich-Weltzien R, Benzian H, Holmgren C, van Palenstein Helderman W. PUFA -an index of clinical consequences of untreated dental caries. Community Dent Oral Epidemiol. 2010;38(1):77-82.
31. Barr DB, Puttaswamy N, Jaacks LM, Steenland K, Rajkumar S, Gupton S, et al. Design and Rationale of the Biomarker Center of the Household Air Pollution Intervention Network (HAPIN) Trial. Environ Health Perspect. 2020;128(4):47010.
32. Rose JE, Levin ED, Benowitz N. Saliva nicotine as an index of plasma levels in nicotine skin patch users. Ther Drug Monit. 1993;15:431–35.
33. Jarvis MJ, Primatesta P, Erens B, Feyerabend C, Bryant A. Measuring nicotine intake in population surveys: Comparability of saliva cotinine and plasma cotinine estimates. Nicotine Tob Res. 2003;5:349–55.
34. McDade TW, Williams S, Snodgrass JJ. What a drop can do: dried blood spots as a minimally invasive method for integrating biomarkers into population-based research. Demography. 2007;44(4):899-925.
35. Barr DB, Kannan K, Cui Y, Merrill L, Petrick LM, Meeker JD, et al. The use of dried blood spots for characterizing children's exposure to organic environmental chemicals. Environ Res. 2021;195:110796.

For peer review only

For peer review only - http://bmjopen.bmj.com/site/about/guidelines.xhtml

36. Yoshida A, Suzuki N, Nakano Y, Kawada M, Oho T, Koga T. Development of a 5' nuclease-based real-time PCR assay for quantitative detection of cariogenic dental pathogens Streptococcus mutans and Streptococcus sobrinus. J Clin Microbiol. 2003;41(9):4438-41.
37. Sharma P, Sane N, Anand SD, Marimutthu P, Benegal V. Assessment of cotinine in urine and saliva of smokers, passive smokers, and nonsmokers: Method validation using liquid chromatography and mass spectrometry. Indian J Psychiatry. 2019;61(3):270-276.
38. Gatzke-Kopp LM, Riis JL, Ahmadi H, Piccerillo HL, Granger DA, Blair CB, Thomas EA. Environmental tobacco smoke exposure is associated with increased levels of metals in children's saliva. J Expo Sci Environ Epidemiol. 2023;33(6):903-910.
39. Aryee M, Jaffe A, Corrada-Bravo H, Ladd-Acosta C, Feinberg A, Hansen K, et al. Minfi: a flexible and comprehensive Bioconductor package for the analysis of Infinium DNA methylation microarrays. Bioinformatics. 2014;30(10):1363–1369.
40. Triche TJ, Weisenberger DJ, Van Den Berg D, Laird PW, Siegmund KD. Low-level processing of Illumina Infinium DNA Methylation BeadArrays. Nucleic Acids Res. 2013;41(7):e90–e90.
41. Stepanov I, Muzic J, Le CT, Sebero E, Villalta P, Ma B, et al. Analysis of 4-hydroxy-1-(3-pyridyl)-1-butanone (HPB)-releasing DNA adducts in human exfoliated oral mucosa cells by liquid chromatography-electrospray ionization-tandem mass spectrometry. Chem Res Toxicol. 2013;26(1):37-45.
42. Chuang CY, Tung JN, Su MC, Wu BC, Hsin CH, Chen YJ, et al. BPDE-like DNA adduct level in oral tissue may act as a risk biomarker of oral cancer. Arch Oral Biol. 2013;58(1):102-9.
43. Paiano V, Maertens L, Guidolin V, Yang J, Balbo S, Hecht SS. Quantitative Liquid Chromatography-Nanoelectrospray Ionization-High-Resolution Tandem Mass Spectrometry Analysis of Acrolein-DNA Adducts and Etheno-DNA Adducts in Oral Cells from Cigarette Smokers and Nonsmokers. Chem Res Toxicol. 2020;33(8):2197-2207.
44. Chen KM, Sun YW, Krebs NM, Sun D, Krzeminski J, Reinhart L, et al. Detection of DNA adducts derived from the tobacco carcinogens, benzo[a]pyrene and dibenzo[def,p]chrysene in human oral buccal cells. Carcinogenesis. 2022;43(8):746-753.
45. McGuffey JE, Wei B, Bernert JT, Morrow JC, Xia B, Wang L, et al. Validation of a LC-MS/MS method for quantifying urinary nicotine, six nicotine metabolites and the minor tobacco alkaloids--anatabine and anabasine--in smokers' urine. PLoS One. 2014;9(7):e101816.
46. van den Hooven EH, Pierik FH, de Kluizenaar Y, Willemsen SP, Hofman A, van Ratingen SW, et al. Air pollution exposure during pregnancy, ultrasound measures of fetal growth, and adverse birth outcomes: a prospective cohort study. Environ Health Perspect. 2012;120(1):150-6.
47. Malmqvist E, Liew Z, Källén K, Rignell-Hydbom A, Rittner R, Rylander L, et al. Fetal growth and air pollution - A study on ultrasound and birth measures. Environ Res. 2017;152:73-80.

For peer review only

48. Iñiguez C, Esplugues A, Sunyer J, Basterrechea M, Fernández-Somoano A, Costa O, et al. Prenatal Exposure to NO2 and Ultrasound Measures of Fetal Growth in the Spanish INMA Cohort. Environ Health Perspect. 2016;124(2):235-42.
49. Aguilera I, Garcia-Esteban R, Iñiguez C, Nieuwenhuijsen MJ, Rodríguez A, Paez M, et al. Prenatal exposure to traffic-related air pollution and ultrasound measures of fetal growth in the INMA Sabadell cohort. Environ Health Perspect. 2010;118(5):705-11.
50. Zhao N, Qiu J, Ma S, Zhang Y, Lin X, Tang Z, et al. Effects of prenatal exposure to ambient air pollutant PM10 on ultrasound-measured fetal growth. Int J Epidemiol. 2018;47(4):1072-1081.
51. Risom L, Møller P, Loft S. Oxidative stress-induced DNA damage by particulate air pollution. Mutat Res. 2005;592(1-2):119-37.
52. IARC Working Group on the Evaluation of Carcinogenic Risks to Humans. Some non-heterocyclic polycyclic aromatic hydrocarbons and some related exposures. IARC Monogr Eval Carcinog Risks Hum. 2010;92:1-853.
53. Puttaswamy N, Saidam S, Rajendran G, Arumugam K, Gupton S, Williams EW, et al. Cross-validation of biomonitoring methods for polycyclic aromatic hydrocarbon metabolites in human urine: Results from the formative phase of the Household Air Pollution Intervention Network (HAPIN) trial in India. J Chromatogr B Analyt Technol Biomed Life Sci. 2020;1154:122284.
54. Thomas JL, Guo H, Carmella SG, Balbo S, Han S, Davis A, et al. Metabolites of a tobacco-specific lung carcinogen in children exposed to secondhand or thirdhand tobacco smoke in their homes. Cancer Epidemiol Biomarkers Prev. 2011;20(6):1213-21.
55. Vardavas CI, Fthenou E, Patelarou E, Bagkeris E, Murphy S, Hecht SS, et al. Exposure to different sources of second-hand smoke during pregnancy and its effect on urinary cotinine and tobacco-specific nitrosamine (NNAL) concentrations. Tob Control. 2013;22(3):194-200.
56. Nikam SS, Gurjar M, Singhavi H, Patil A, Singh A, Villalta P, et al. Simultaneous analysis of urinary total 4-(methylnitrosamino)-1-(3-pyridyl)-1-butanol, N'-nitrosonornicotine, and cotinine by liquid chromatography-tandem mass-spectrometry. Sci Rep. 2021;11(1):20007.
57. Ji SQ, Han R, Huang PP, Wang SY, Lin H, Ma L. Iron deficiency and early childhood caries: a systematic review and meta-analysis. Chin Med J (Engl). 2021;134(23):2832-2837.
58. Li Z, Wei X, Shao Z, Liu H, Bai S. Correlation between vitamin D levels in serum and the risk of dental caries in children: a systematic review and meta-analysis. BMC Oral Health. 2023;23(1):768.
59. Eyles D, Anderson C, Ko P, Jones A, Thomas A, Burne T, et al. A sensitive LC/MS/MS assay of 25OH vitamin D3 and 25OH vitamin D2 in dried blood spots. Clin Chim Acta. 2009;403(1-2):145-51.
60. Blackburn EH. Structure and function of telomeres. Nature. 1991;350(6319):569-73.
61. Theall KP, McKasson S, Mabile E, Dunaway LF, Drury SS. Early hits and long-term consequences:tracking the lasting impact of prenatal smoke exposure on telomere length in children. Am J Public Health. 2013;103(Suppl 1):S133–35.

For peer review only

62. Lu L, Johnman C, McGlynn L, Mackay DF, Shiels PG, Pell JP. Association between exposure to second-hand smoke and telomere length: cross-sectional study of 1303 non-smokers. Int J Epidemiol. 2017;46(6):1978-1984.
63. Langer EK, Johnson KJ, Shafer MM, Gorski P, Overdier J, Musselman J, et al. Characterization of the elemental composition of newborn blood spots using sector-field inductively coupled plasma-mass spectrometry. J Expo Sci Environ Epidemiol. 2011;21(4):355-64.
64. Williams SR, McDade TW. The use of dried blood spot sampling in the national social life, health, and aging project. J Gerontol B Psychol Sci Soc Sci. 2009;64(Suppl 1):i131-6.
65. Ward JH. Hierarchical grouping to optimize an objective function. J Am Stat Assoc. 1963;58(301):236-244.
66. Keil AP, Buckley JP, O'Brien KM, Ferguson KK, Zhao S, White AJ. A quantile-based g-computation approach to addressing the effects of exposure mixtures. Environ Health Perspect. 2020;128(4):47004.
67. R Core Team. R: A language and environment for statistical computing [Internet]. Vienna, Austria: R Foundation for Statistical Computing; 2015.
68. Huber W, Carey VJ, Gentleman R, Anders S, Carlson M, Carvalho BS, et al. Orchestrating high-throughput genomic analysis with Bioconductor. Nat Methods. 2015;12(2):115–121.

Contributors - NP, SP and AC conceptualised the project and were involved in developing study methodology and funding acquisition. SP and NP drafted the manuscript. AC, DBB, SG and KB edited the manuscript contributing critical content expertise.

Funding - The CARES study is supported by the Fogarty International Center (D43TW010540) with participation from the National Institute of Dental and Craniofacial Research. The HAPIN trial is funded by the U.S. National Institutes of Health (cooperative agreement 1UM1HL134590) in collaboration with the Bill & Melinda Gates Foundation (OPP1131279).

Competing interests - None declared.

Provenance and peer review - Not commissioned; externally peer reviewed.

Data sharing statement - The data utilised for this study are not publicly available but de-identified data may be available on request, subject to approval by the internal review board and under a formal data use agreement. Contact the corresponding author for more information.

For peer review only

Table 1: List of biomarkers and summary of analysis scheme planned in the CARES - HAPIN study

**Biomarker**	**Matrix**	**Subject**	**Informs about**	**Method**	**Reference**
COT, 3HC	Saliva and Urine	Child/Mother	Recent exposure to ETS	LC-ESI-MS/MS	Sharma et al. 2019;[37] McGuffey et al. 2014 [45]
Metals	Saliva and DBS	Child	Exposure to ETS	QQQ-ICP-MS	Gatzke-Kopp et al. 2023;[38] Langer et al. 2011 [63]
Malondialdehyde	Saliva	Child/Mother	Oxidative damage to membranes	Immunoassay	ELISA kit
Uric acid	Saliva	Child/Mother	Cellular metabolism	Immunoassay	ELISA kit
Total antioxidant capacity	Saliva	Child/Mother	Antioxidant capacity	FRAP	ELISA kit
Reduced Glutathione	Saliva	Child/Mother	Antioxidant capacity	Immunoassay	ELISA kit
Streptococcus Mutans, Lactobacilli	Saliva	Child/Mother	Cariogenic bacterial load	qRT-PCR	Yoshida et al. 2003 [36]
DNA Methylation	Buccal cells	Child	Epigenetic modification	BeadArray	Triche et al. 2013 [40]
DNA Adduct	Buccal cells	Child	DNA damage	LC-ESI-MS/MS	Stepanov et al. 2013;[41] Chuang et al. 2013 [42]
8OHdG	Urine	Child	Damage to DNA base pair	LC-ESI-MS/MS	McGuffey et al. 2014 [45]
PAH metabolites	Urine	Child	Exposure to PAHs	HPLC-FLD	Puttaswamy et al. 2020 [53]
NNAL	Urine	Child	Exposure to carcinogenic TSNAs	LC-ESI-MS/MS	Nikam et al. 2021 [56]
25 hydroxyvitamin D2 and 25 hydroxyvitamin D3	DBS	Child	Vitamin D reserve	LC-ESI-MS/MS	Eyles et al. 2009 [59]
Telomere Length	DBS	Child	Cellular ageing	qPCR assay kit	ScienCell, CA, US

For peer review only - http://bmjopen.bmj.com/site/about/guidelines.xhtml

Figure Legends:

Figure 1. Conceptual framework of the Caries Risk from exposure to Environmental tobacco Smoke - Household Air Pollution Intervention Network (CARES - HAPIN) study

Figure 2. General mechanism linking ETS exposure and ECC occurrence

For peer review only

For peer review only - http://bmjopen.bmj.com/site/about/guidelines.xhtml

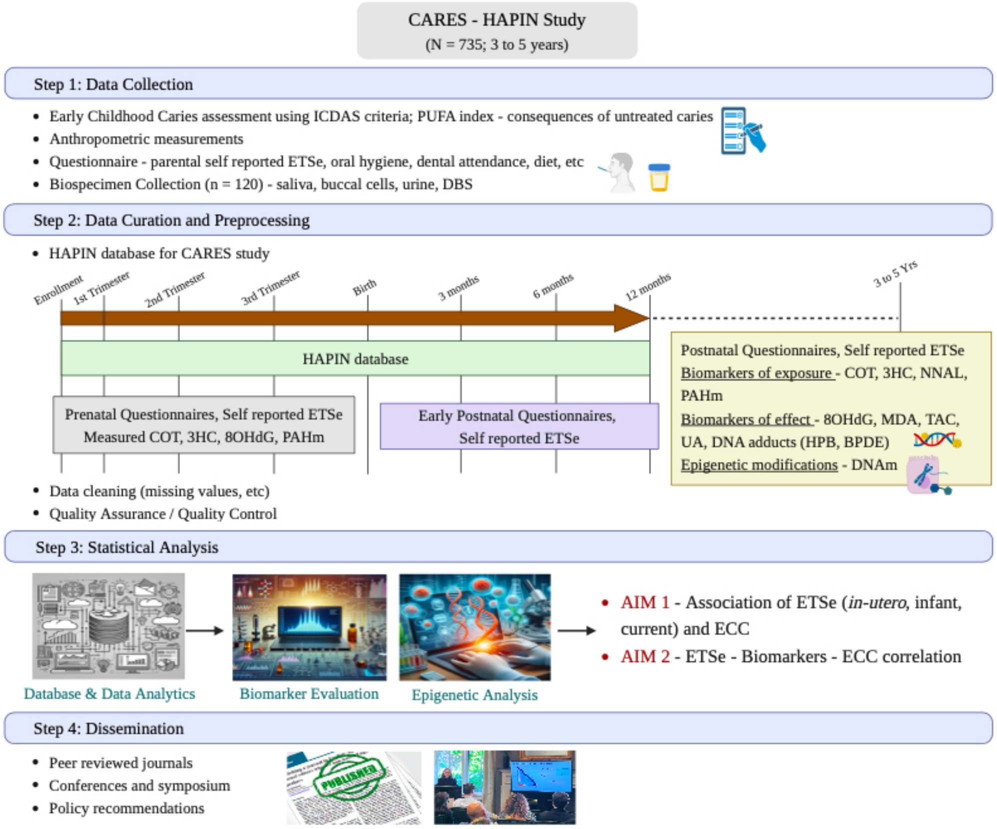

Conceptual framework of the Caries Risk from exposure to Environmental tobacco Smoke - Household Air Pollution Intervention Network (CARES - HAPIN) study

16x14mm (1200 x 1200 DPI)

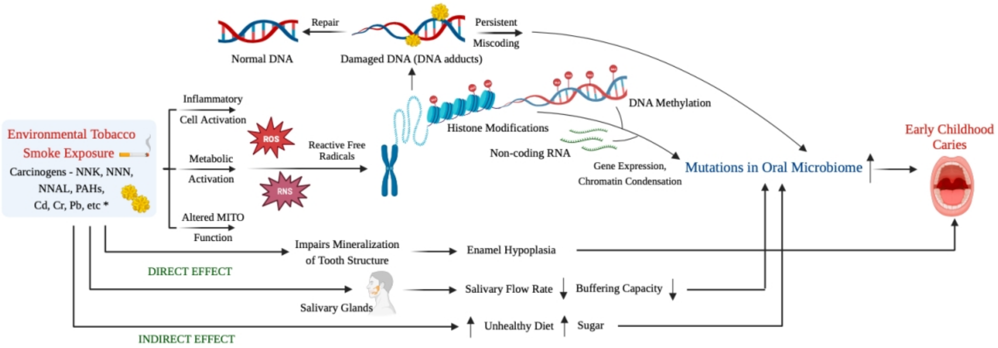

General mechanism linking ETS exposure and ECC occurrence

30x10mm (1200 x 1200 DPI)
